# A Critical Cybersecurity Analysis and Future Research Directions for the Internet of Things: A Comprehensive Review

**DOI:** 10.3390/s23084117

**Published:** 2023-04-19

**Authors:** Usman Tariq, Irfan Ahmed, Ali Kashif Bashir, Kamran Shaukat

**Affiliations:** 1 Management Information System Department, College of Business Administration, Prince Sattam Bin Abdulaziz University, Al-Kharj 16278, Saudi Arabia; 2 Department of Computer Science, College of Engineering, Virginia Commonwealth University, Richmond, VA 23284, USA; iahmed3@vcu.edu; 3 Department of Computing and Mathematics, Manchester Metropolitan University, Manchester M156BH, UK; a.bashir@mmu.ac.uk; 4 School of Information and Physical Sciences, The University of Newcastle, Newcastle 2308, Australia; kamran.shaukat@uon.edu.au

**Keywords:** IoT security, data security, network security, anomaly detection and countermeasures

## Abstract

The emergence of the Internet of Things (IoT) technology has brought about tremendous possibilities, but at the same time, it has opened up new vulnerabilities and attack vectors that could compromise the confidentiality, integrity, and availability of connected systems. Developing a secure IoT ecosystem is a daunting challenge that requires a systematic and holistic approach to identify and mitigate potential security threats. Cybersecurity research considerations play a critical role in this regard, as they provide the foundation for designing and implementing security measures that can address emerging risks. To achieve a secure IoT ecosystem, scientists and engineers must first define rigorous security specifications that serve as the foundation for developing secure devices, chipsets, and networks. Developing such specifications requires an interdisciplinary approach that involves multiple stakeholders, including cybersecurity experts, network architects, system designers, and domain experts. The primary challenge in IoT security is ensuring the system can defend against both known and unknown attacks. To date, the IoT research community has identified several key security concerns related to the architecture of IoT systems. These concerns include issues related to connectivity, communication, and management protocols. This research paper provides an all-inclusive and lucid review of the current state of anomalies and security concepts related to the IoT. We classify and analyze prevalent security distresses regarding IoT’s layered architecture, including connectivity, communication, and management protocols. We establish the foundation of IoT security by examining the current attacks, threats, and cutting-edge solutions. Furthermore, we set security goals that will serve as the benchmark for assessing whether a solution satisfies the specific IoT use cases.

## 1. Introduction

The Internet of Things (IoT) has emerged as a critical technology in modern society, with significant implications for cybersecurity. IoT devices are ubiquitous, interconnected, and often lack essential security features, leaving them vulnerable to a range of cyber threats. Malicious actors can exploit these vulnerabilities to gain sensitive data, launch distributed denial-of-service (DDoS) attacks, and even take control of critical infrastructure. A large-scale cyber-attack on IoT networks could have severe consequences, including disrupting essential services and causing widespread economic damage.

The IoT is a complex network of interconnected devices and individuals collaborating to monitor and exchange information about their usage and environmental conditions. The system consists of smart devices equipped with embedded systems comprising CPUs, sensors, and connectivity hardware that collect, transmit, and respond to information obtained from their surroundings within the IoT ecosystem. These IoT devices communicate with an IoT gateway or another edge device to exchange sensor data with each other. The data is either transferred to the cloud for analysis or processed locally. Periodically, these devices exchange information and take appropriate actions based on that exchange. In most cases, IoT devices function autonomously without any human intervention. The IoT is a rapidly expanding field with unique challenges regarding device interoperability, data privacy, and security.

IoT not only enables individuals to live and work more efficiently and gives them greater control over their lives. Businesses rely heavily on the IoT because it provides technological devices for automating enterprise environments. With IoT, organizations can obtain real-time insights into how various systems function, allowing them to optimize processes and reduce labor costs. Furthermore, IoT provides visibility into business transactions, reduces manufacturing and shipping costs, and enhances service efficiency. Intelligent IoT apps, also known as prefabricated SaaS systems, are equipped with machine learning techniques to analyze vast volumes of data collected from interconnected sensors, providing corporate users with actionable insights via interfaces. By monitoring KPIs (key performance indicators), MTBF (mean time between failure) rates, and other metrics in real-time, IoT dashboards and alerts can help detect irregularities and initiate automatic repairs or preventative actions.

One of the biggest obstacles the IoT brings is ensuring its security. These devices gather sensitive data, such as what you say and do at home and the workplace. Users’ trust in the IoT hinges on its dependability, yet it has a dismal track record of securing data. Many connected systems fail to adequately safeguard users’ and device data by mismanaging it while it is stored and in transit. Even in well-established programs, software vulnerabilities are constantly being uncovered, yet many IoT devices cannot be updated, leaving them permanently vulnerable. Due to their intrinsic lack of protection, IoT devices such as routers and cameras are increasingly targeted by hackers who exploit them as part of massive, interconnected botnets. According to estimates from technology analyst firm IDC (International Data Corporation) [1], IoT devices will produce 79.4 zettabytes of data in the next five years. Some of this Internet of Things data will be “compact and anomalous”, as predicted by IDC. This means it will only consist of relatively short updates, such as those provided by sensors or smart meters. In addition, devices such as security cameras with built-in computer vision might produce massive amounts of data. According to IDC’s forecasts, the amount of data generated by Internet of Things (IoT) devices will skyrocket in the following years. The report claims that while video surveillance is the current leader in data production, other industries and medical applications will soon overtake it. Interconnected drones with built-in cameras were also projected to become an important data collection tool. Data from a wide variety of sensors, including audio, video, and specialized automotive sensor data, will be generated by autonomous vehicles in the near future.

Conducting a thorough vulnerability investigation [2] is the first step in creating an IoT-enabled environment. This involves looking at the devices, protocols, and user/customer backends of the infrastructure to identify possible weak points. Risk management and assessment must be performed throughout the IoT implementation lifecycle, especially when the deployment is larger or spans more regions. Due to the many data formats and processing capabilities of IoT devices, there is no “one size fits all” cybersecurity solution that can protect any Internet of Things deployment.

**Figure 1:**
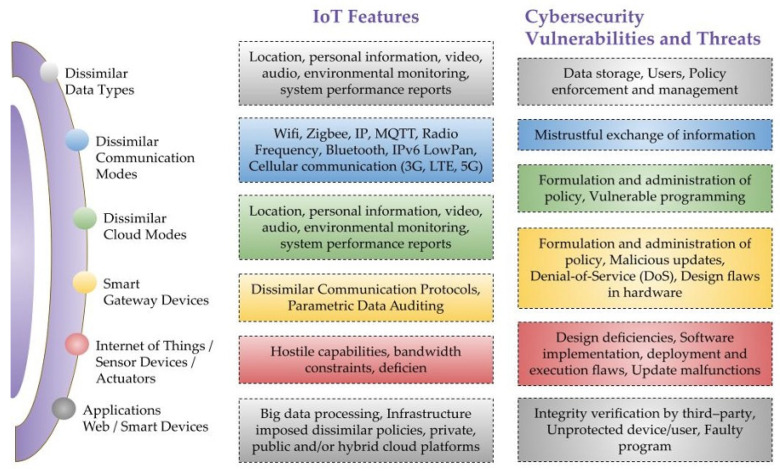
IoT Security Considerations (Inspiration for Figure 1 was inherited from: Building trust in IoT devices with powerful IoT security solutions. (Telit-Cinterion). Thales Group. https://www.thalesgroup.com/en/markets/digital-identity-and-security/iot/iot-security (accessed on 5 February 2023)).

Figure 1 shows that most IoT solutions are reasonably priced and aimed toward the consumer market, with little thought given to issues such as security and privacy. Cybercriminals are eager to take advantage of such vulnerabilities by adding them to a botnet or exploiting them to spy on their owners. As a result, we must take measures to safeguard this technology. In addition, the urgency of this need is only going to rise as the number of available IoT devices continues to proliferate. Due to limited capacity and varied design, IoT devices are open to various security risks. Threats to wireless ad hoc networks increase when devices are deployed to uncontrolled and potentially dangerous environments. It is common in hetnets [3] to experience attacks such as sinkholes, blackholes, wormholes, sybils, denial-of-service (DoS), node capture, and node injection.

During our literature review, we were able to identify several key research gaps in securing the IoT, such as:

1. There is a lack of comprehensive security solutions for IoT devices. The number of devices and the different applications that they are used for having resulted in a complex ecosystem that is hard to secure. This complexity is exacerbated by the limited computational resources of many IoT devices, which makes it difficult to implement advanced security solutions.
2. There is a lack of protocol standardization in IoT device security. The lack of universal security measures makes it easier for attackers to exploit vulnerabilities, resulting in a higher risk of security breaches. Without a common taxonomy, it becomes difficult to ensure the security of an IoT ecosystem, which consists of several different types of devices, communication protocols, and applications.
3. There is a need for research to identify new security risks that arise from the integration of IoT devices with other systems, such as layer-based services. The integration of IoT devices with diverse systems introduces new vulnerabilities, which need to be identified and addressed.
4. There is a need for more research on access control driven anomalies and countermeasures related to IoT devices. The massive deployment of these devices has led to the collection of vast amounts of data, and it is essential to ensure that this data is protected from unauthorized access and misuse.

**Figure 2:**
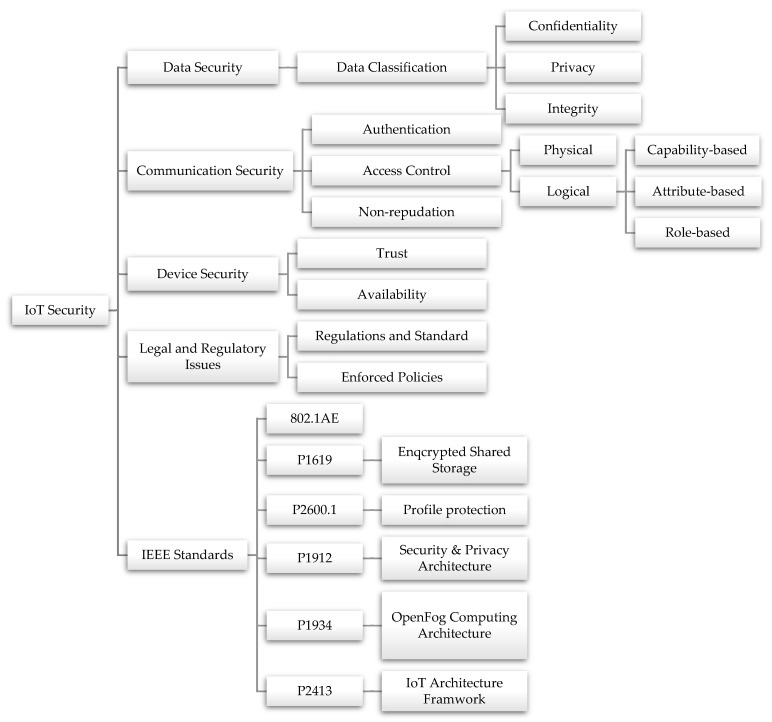
Basic security taxonomy for the IoT.

To ensure the security of private information, it is necessary to safeguard the data collected by IoT devices, as depicted in Figure 2. Additionally, it is important to protect the communication between these devices to prevent unauthorized interception of data. In some cases, IoT settings involve physical entities exchanging data to provide users with relevant information. This highlights the criticality of securing these devices. The potential security threats associated with the Internet of Things can have severe consequences, including data breaches, loss of sensitive information, and compromised personal privacy. Thus, addressing the security concerns related to the IoT is vital to ensure its safe and efficient operation.

We acknowledge that IoT security vulnerabilities are vast & complex and covering all of them in a research paper is not always feasible. In this regard, we want to point out that the selection of security vulnerabilities discussed in our research paper is based on various factors, such as the research objectives, the scope of the study, the availability of data, and the relevance to the research question.

We acknowledge that other security vulnerabilities related to the IoT exist, and are also important to address. However, the inclusion of all IoT security vulnerabilities in the paper would have been beyond the scope of the research, and it may have led to an overly broad and unfocused discussion. Therefore, we focused on specific security vulnerabilities that were most relevant to the research question and objectives while acknowledging that other vulnerabilities exist in the IoT ecosystem.

In order to discern dissimilarities among the published literature, we have taken into account several factors, including the range of the literature; research methods employed; level of scrutiny (i.e., methodology, outcomes, and limitations); reliability of sources (such as academic journals, white papers, and industry publications); a preferred timeframe (from 2019 to February 2023); and a particular focus has been given to literature that highlights gaps in current research with pragmatic suggestions for enhancing cybersecurity within IoT systems.

In this paper, the remaining content is structured as follows: In Section 2, the general design, components, and protocols for the Internet of Things are discussed. Section 3 focuses on potential security concerns with the IoT. Section 4 introduces the unique aspects of IoT anomaly detection compared to IT security. Section 5 explains the classification of IoT Access Control. Section 6 presents our perspective on how artificial intelligence (AI) and machine learning (ML) can impact IoT security. Lastly, in Section 7, this review paper is concluded.

## 2. General Design, Components, and Protocols for the Internet of Things

**Figure 3:**
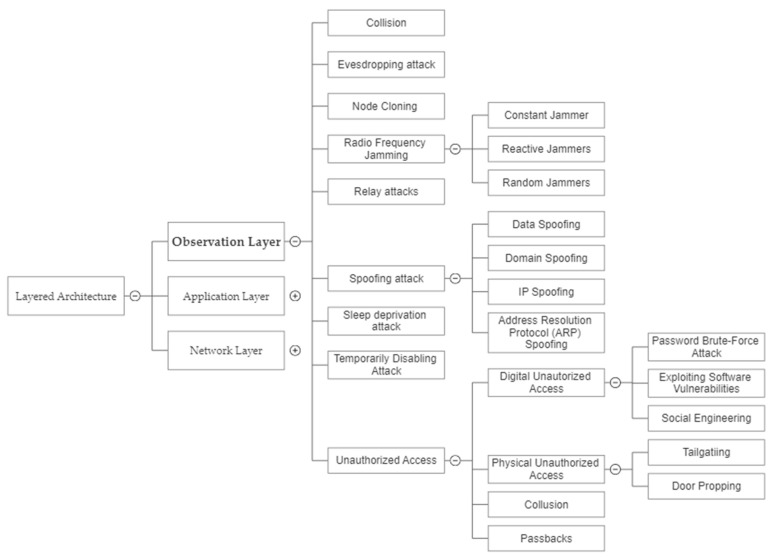
IoT risk taxonomy at the observation layer.

Even though there is no set template for IoT design, it often consists of three distinct but interconnected layers: observation, network, and application. However, CISCO, a digital communication corporation, took it a step farther and added a total of seven levels [4]. CISCO defined layers were (1) physical devices, (2) communication and processing, (3) data analysis and transformation, (4) data storage, (5) data aggregation and abstraction, (6) application, and (7) collaboration and business process. As per Figure 3, we have focused on the security vulnerabilities which concern observation, network, and application layers. A multi-layered protection method aims to ensure that every component of the IoT cybersecurity strategy has a backup to compensate for any weaknesses or shortcomings. Together, these layers strengthen enterprise defenses and lay the framework for an effective cybersecurity strategy for the Internet of Things (IoT). Furthermore, we have also examined the National Institute of Standards and Technology (NIST) Cybersecurity Framework [5], which is based on primary functions, such as Identify, Protect, Detect, Respond, and Recover.

We implemented a “Tuple Model” to create a threat scenario for IoT security. The model consisted of the attacker, the attack vector, the target, and/or the impact. The attacker element identified the entity or group responsible for the attack, while the attack vector element described the method or means used to execute the attack. The target element represented the specific IoT device or system under attack, while the impact element described the potential consequences or damage resulting from the attack.

This Tuple Model provided a framework for capturing the diversity of threats and vulnerabilities in an IoT system and facilitated the development of a comprehensive threat model that accounted for all possible scenarios. Consequently, this model could be expanded to include additional elements to capture more complex threats, such as multi-stage attacks or those that involve multiple targets.

### 2.1. Observation Layer

A variety of IoT sensors and other hardware components make up the observation layer, which is also in charge of data exchange and gathering. Sensors and other technologies detecting and relaying information are crucial to the Internet of Things. [6]. A WSN uses a network of wirelessly connected, intelligent sensors to collect information about environmental conditions. Sensitive information is sent to a central hub or base station via a single or several relay stations.

Figure 3 provides a comprehensive and systematic analysis of security threats at the observation layer of the IoT. The figure identifies various categories of potential security threats at this layer, including physical, device, and data security. By breaking down the potential threats into these categories, the figure offers a clear and organized overview of the risks that need to be addressed when securing the observation layer of the IoT.

### 2.2. Network Layer

With the help of the observation layer’s input, the network layer stores or transmits the gathered data to the application layer for further processing. When it comes to the context of the Internet of Things, this layer is the most crucial, since it unifies the numerous forms of communication technology that make it possible for IoT devices to communicate with one another. Among the most popular of these communication methods are ZigBee [7], BLE (i.e., piconet) [8,9], 6LoWPAB [10,11,12], LoRaWAN [13,14,15], and readable tags.

The criteria for wireless technologies driven by use cases (i.e., applications) and the Internet of Things architecture are outlined in Table 1. This paper mainly focuses on IoT security, which can have overlapping concepts with classical wireless networks. While some of the content can be applied to both, there are differences between IoT devices and classical wireless network devices. IoT devices are often low capability, have limited power sources, generate large volumes of data, and can be left unattended for extended periods. Therefore, it is crucial to consider the unique characteristics of IoT devices when designing security measures. On the other hand, classical wireless networks and devices may have different requirements and capabilities. While some security principles may apply across both types of networks, the specific implementation and considerations can differ. Therefore, the paper may have emphasized the need to address IoT security threats explicitly that can be overlapping to the broader context of wireless networks. There are several similarities in the protocols and standards used in both IoT and classical wireless networks. For example, both use the IEEE 802.11 standard for wireless LANs, and both use the Transmission Control Protocol/Internet Protocol (TCP/IP) for communication. Both also use security protocols such as Wi-Fi Protected Access (WPA) and Transport Layer Security (TLS) to protect data transmission. However, there are also some differences in the protocols and standards used in the two types of networks. For instance, IoT devices often use low-power, low-data-rate wireless technologies (i.e., explained in Table 1. Assessment of Wireless IoT Technologies) such as Zigbee or Bluetooth Low Energy (BLE), whereas classical wireless networks tend to use high data rate protocols such as long-term evolution (LTE) or WiMAX. Additionally, IoT devices often have limited processing power and memory, which affects the type of protocols and standards that can be used. Therefore, specialized protocols such as Constrained Application Protocol (CoAP) and Message Queuing Telemetry Transport (MQTT) are frequently used in IoT devices. Specific criteria were given attention for both local and remote scenarios, including Internet of Things gateways, wireless device connectivity, hardware connectors, and communication protocols.

**Figure 4:**
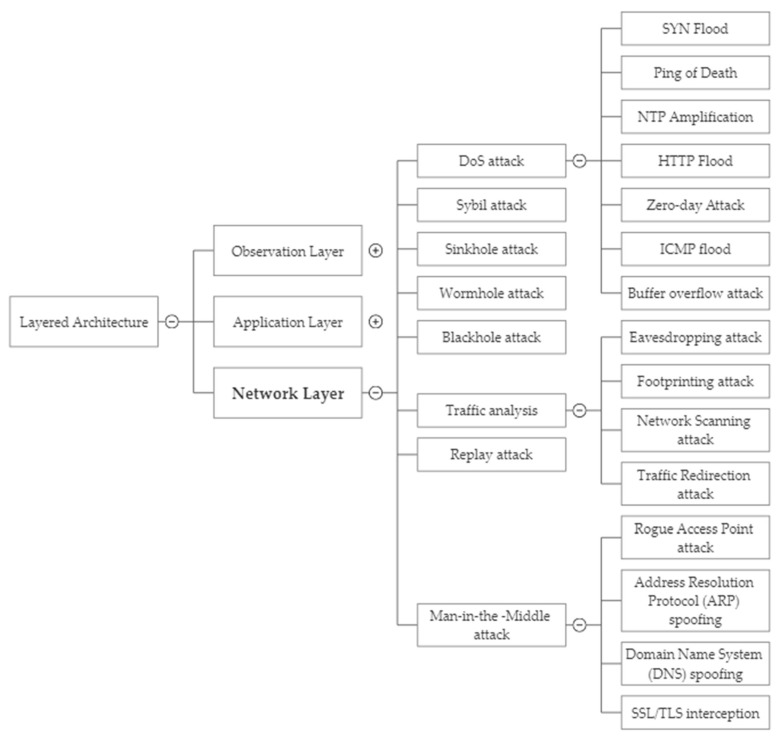
IoT risk taxonomy at the network layer.

Figure 4 offers a comprehensive analysis of the security threats that are specific to the network layer of IoT. The figure is a useful tool for understanding and visualizing the complex security issues at this layer, including various network protocols, communication technologies, and architectures. The taxonomy is organized into several categories, such as network protocol vulnerabilities, network infrastructure vulnerabilities, and malicious network traffic, and provides a detailed breakdown of the different types of threats that can occur in each category. This systematic approach helps to identify potential vulnerabilities in the network layer of IoT, which can inform the development of effective security measures to mitigate these risks.

### 2.3. Application Layer

When it comes to interoperability between IoT devices and their communication networks, the application layer is the key. It mediates between the activities of an IoT device and the subsequent transfer of data to the network in a usable manner. Several considerations, including the type of device and the task it will carry out, determine the optimal protocol for every given Internet of Things application. Constraint Application Protocol (CoAP) [16,17,18] and Message Queueing Telemetry Transport (MQTT) [19,20,21] are the most often used application protocols. CoAP was developed to let low-power, low-resource devices connect to the Internet of Things via slow, unreliable networks. Its primary use is in M2M (machine-to-machine) systems, which facilitates communication between machines to carry out tasks such as metering and controlling HVAC (heating, ventilation, and air conditioning) systems and smart lighting. Information transfer in CoAP occurs via UDP (User Datagram Protocol). CoAP uses the encryption and authentication features of UDP to keep data safe. CoAP employs Datagram TLS (Transport Layer Security) over UDP in the same way that HTTP (Hypertext Transfer Protocol) employs TLS over TCP (Transmission Control Protocol). RSA (Rivest-Shamir-Adleman) [22], AES (Advanced Encryption Standard) [23,24,25], and other ciphers are all supported by DTLS (Datagram Transport Layer Security).

MQTT was created as a minimalist publish/subscribe message system, ideally suited for establishing connections between remote devices with a low resource need and limited bandwidth. MQTT is an excellent option for wireless networks with variable latency due to periodic throughput restrictions or unstable connectivity. TLS security-enabled MQTT protocol can support bi-direction communication among millions of devices in a reliable, lightweight, and efficient manner [26,27,28]. An investigation has revealed the following differences between MQTT and CoPA that should be considered while implementing IoT technologies:

(a) MQTT is a TCP-based protocol, while CoAP is an HTTP-based protocol. CoAP can also use UDP for more efficient communication, making it a good fit for low-power, lossy networks. REST is an architectural style for building web services and is often used in conjunction with CoAP to provide a standardized interface for web-based applications to interact with IoT devices.
(b) MQTT uses a topic-based approach for message routing, where messages are published to a topic and subscribers can subscribe to specific topics to receive messages. CoAP/REST uses URIs (Uniform Resource Identifiers) to identify resources, which can be manipulated using standard HTTP methods such as GET, PUT, POST, and DELETE.
(c) MQTT provides three QoS levels, while CoAP/REST provides four. MQTT QoS levels range from 0 to 2, with 0 providing no guarantees and 2 providing the highest level of reliability. CoAP/REST QoS levels range from 0 to 3, with 0 providing no guarantees and 3 providing the highest level of reliability.
(d) MQTT supports bidirectional communication between clients and servers, while CoAP/REST supports both unidirectional and bidirectional communication.
(e) MQTT has basic security features, such as username and password authentication, but it lacks more advanced security features such as message encryption and authorization. On the other hand, CoAP/REST provides more comprehensive security features such as message integrity, confidentiality, and authentication using Datagram Transport Layer Security (DTLS).
(f) MQTT has a larger maximum payload size than CoAP/REST, making it a better option for applications requiring large data transfer. CoAP/REST is designed for constrained environments and has a smaller maximum payload size.
(g) MQTT provides higher reliability than CoAP/REST, particularly at higher QoS levels. MQTT QoS level 2 provides assured message delivery, while CoAP/REST only provides a best-effort approach.
(h) MQTT is a standardized protocol that is widely used in the IoT industry. CoAP/REST, while also a standard protocol, is not as widely used in the IoT industry and is more commonly used in the machine-to-machine (M2M) communications domain.
(i) MQTT has a lower overhead than CoAP/REST due to its simpler message format and smaller header size. This makes it a better option for applications that require low-latency communication and efficient use of network resources.

**Figure 5:**
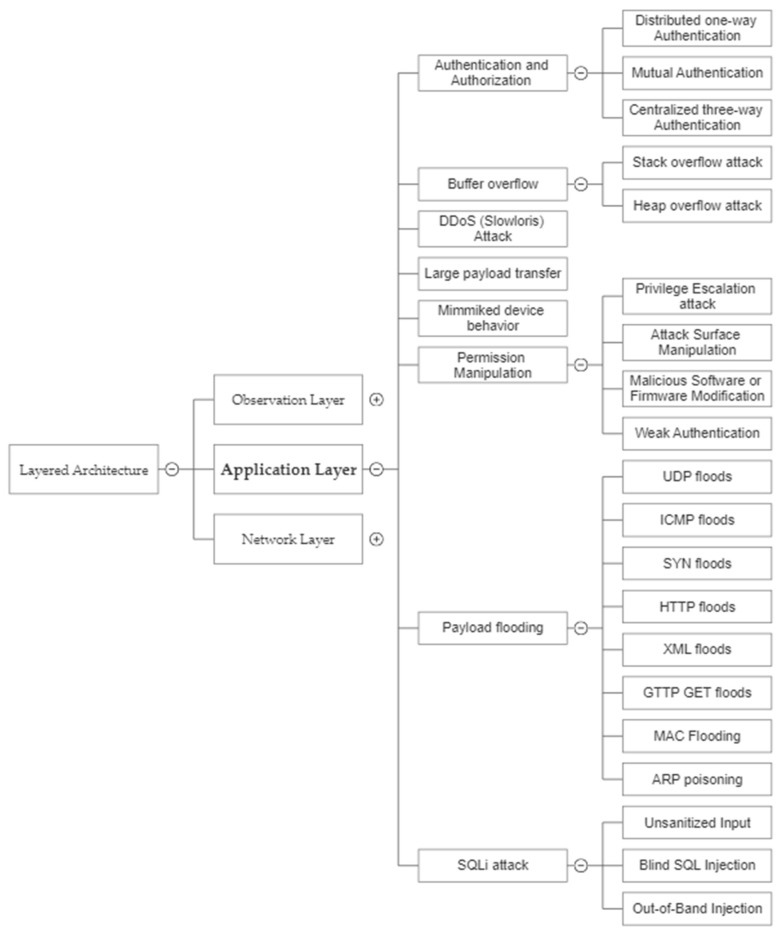
IoT risk taxonomy at the application layer.

Figure 5 serves as a comprehensive analysis of security risks and threats associated with the application layer of the Internet of Things architecture and is structured logically and systematically, thereby facilitating a more thorough understanding of the nature and complexity of these potential security issues.

## 3. Potential Security Issues with the Internet of Things

Most Internet-enabled gadgets are not built with security in mind. There are, therefore, many inherent risks to the safety of the IoT, some of which can be catastrophic. IoT security has a paucity of defined standards and regulations compared to other technical solutions. What is more, most individuals are unaware that their IoT devices put them at risk. Some security challenges afflicting the IoT include a lack of transparency, insufficient security integration, vulnerabilities in open-source code, unpatched vulnerabilities, insecure APIs (application programming interfaces), and insufficient testing.

### Data, Network and Device Security

Cybersecurity must be in place at every stage of the lifecycle of an IoT-interconnected environment to protect that infrastructure from unauthorized access, alteration, or loss. It is a plan of action that consider not only the technological safeties such as firewalls and virus protection but also the user protections such as proper authentication and access limitations and logical safeguards such as encryption and secure programming for applications.

Table 2 provides a comprehensive overview of the security challenges in each layer and proposes suitable solutions to mitigate these risks. The critical analysis of research questions (i.e., attack, portrayal, and purpose) and the extensive review of related literature provides the research community with valuable insights and guidance in navigating the vast IoT security landscape.

Thus, in context of Table 2, it is evident that the security flaws in the IoT may very well have devastating effects on businesses and individuals alike. Data theft, cyber-attacks, and the suspension of essential services are all possible results of a vulnerability being exploited. In addition to the risk of sanctions and legal action, security flaws can increase expenses for the companies employing the device. Additionally, hackers can use security flaws in IoT devices to access and change the data being transferred from these devices in the real world. Last, but not least, security flaws can be exploited to cause malfunctions or even total shutdowns of devices or networks.

## 4. What Distinguishes IoT Anomaly Detection from IT Security?

The IoT infrastructure can vary widely depending on the specific use case and requirements. However, in general, a typical IoT infrastructure may include hardware, software, and network components such as:

(a) Hardware:
  - Microcontroller or microprocessor with low power consumption and wireless connectivity (e.g., Wi-Fi, Bluetooth, and Zigbee);
  - Sensors and actuators for data acquisition and control;
  - Power source (e.g., battery, energy harvesting, and power adapter);
  - Memory and storage for data and software;
  - Security features (e.g., secure boot, encryption, and access control).
(b) Network:
  - Wireless communication protocols (e.g., Wi-Fi, Bluetooth, Zigbee, and LoRaWAN);
  - Network topology (e.g., star, mesh, and point-to-point);
  - Gateway or edge device for data aggregation and processing;
  - Cloud or server infrastructure for data storage and analysis;
  - Security protocols (e.g., SSL/TLS, VPN, firewalls, and intrusion detection/prevention).
(c) Software:
  - Operating System (e.g., embedded Linux, FreeRTOS, and Zephyr);
  - Middleware (e.g., MQTT, CoAP, and AMQP);
  - Database (e.g., Apache Cassandra, MongoDB, and InfluxDB);
  - Application Development Tools (applied) (e.g., software development kits (SDKs) and integrated development environments (IDEs).

In consideration with benchmarking details of applied hardware, network and software specifications, when compared to standard IT security technologies such as firewalls, IDSs, and Data Security and Consequence Administration Procedure (DSCAP), the idea of anomaly discovery is very distinct both technologically and in terms of the fundamental monitoring strategy.

As seen in Table 3, detecting anomalies requires looking within, as well as outside of, a network. Anomaly detection is an alternative method of security to the more conventional firewalls and intrusion detection systems. When deciding whether a certain action poses a risk, both rely on the latest security updates released by IT security service providers. As a direct consequence, current strategies for protection are continually falling behind potential attackers. As the so-called blacklists maintained by security entities are never updated until after an attack has already taken place. This kind of patching leaves networks vulnerable to intrusion, especially in the IoT/IIoT/IoMT industry, where updates are often delayed preventing disruption and failures.

Consequently, authenticating IoT devices is crucial to establishing confidence that the devices being linked are in fact what they claim to be. Therefore, effective access control can regulate who can access and utilize what resources and under what conditions.

## 5. Classification of Internet of Things Access Control

The term “access control” describes a class of security mechanisms that restrict users’ permission to access particular components of a networked computing and sensing environment. It is a fundamental part of any governance system designed to protect an organization’s or a municipality’s Internet of Things infrastructure from outside threats. Access control can be physical or logical, depending on the system’s needs being protected. An IoT-enabled access control system is a useful tool for ensuring the safety of buildings, classrooms, dormitories, and intangible technological assets such as servers and computer equipment. Logical access control is a method of controlling who may access what is on a network, including computers, servers, and data. Logical access control systems examine multiple identifiers such as passwords, PINs, biometric scans, cryptographic keys, etc. to verify and provide access to the appropriate individuals or entities. Today’s complex IoT landscapes, which include on-premises and cloud-based resources, make it difficult to manage an access control system.

To function, access controls must first determine the identity of the requesting device or entity, verify that the requesting process or service is indeed who it claims to be, and then allow the requesting account or IP address the privileges and access rights corresponding to that identity. Easy-to-Use Reference File systems and procedures such as Access Protocol and Security Assertion Markup Language are only two examples of the kinds of things that do give access controls by verifying and approving users and organizations before providing them access to systems and operations. Conclusively, it is worth highlighting variety of access control systems available, and it is not uncommon for them to be used in tandem with one another as part of an enterprise’s identity and access management (IAM) framework. Applications can be installed locally, on the cloud, or in both scenarios. To varying degrees, it may prioritize either the internal or external access management of an IoT network’s user base.

As highlighted in Table 4, in order to determine whether a user should be granted access, attribute-based access control (ABAC) looks at the user’s characteristics instead of their behavior. The fundamental benefit of ABAC is its ease of use, since the underlying technical permission settings can be masked behind user profiles, which can be modified by anyone possessing the appropriate rights and still ensure the user’s desired degree of access provided their attributes are true. Rouhani et al. [114] presented a method that offers a degree of transparency to the extent that those requesting access to a resource and the administrators of that asset may both advantage from using it. The technique that had been proposed presents a system architecture with an application that was predicated on Hyperledger Fabric. This technology achieves a high degree of effectiveness while maintaining a low level of computation complexity. The viability of the suggested approach was demonstrated through the examination of a use case involving separate digital libraries. Unfortunately, authors failed to address the coefficient of decentralization aspect of futuristic systems. When the Internet of Things infrastructure spans more than one point and a portion or all those spots run their own identity management, then the IoT infrastructure is said to have decentralized access permissions. Such an environment does not have a single, consistent policy and mechanism for controlling access to the network. If applied access control is not centralized, administration, management, and adherence will all be more difficult to manage, which is why it is so important to have logically centralized control.

Li et al. [115] highlighted the ability of CP-WABE to safeguard information security on the Internet of Health Things (IoHT) as a crucial component of a highly appreciated security technique for attaining flawless access control. To tackle this limitation, a novel method of formulating access policies was devised that makes use of 0–1 coding technology. The strategy’s proponents argue that it can be used to design a powerful and adaptable CP-WABE for the IoHT. In the projected method, contextual circumstances could enhance each rule’s preset building elements, known as strategy bits, which were connected with agents and other functional restrictions. The applied steps of reform were: (a) defining rules for adopters, hosts, applications, and other entities, as well as activities and resources, (b) forming strategies with reusable building blocks called “regulatory elements”, and (c) “governance modules”. Access control was tested and found equally effective in firewall, server, application, database, and data layer.

Song et al. [116], introduced an innovative IoT management approach that leverages distributed ledger technology to aid organizations in setting up an effective supply chain. The design relies on a peer-to-peer fallback procedure, a private data segregation and communication approach, and a security token-based authentication and authorization system. The access control system was divided into two subsystems: the registrar and the inspection. The primary responsibility of the registrar’s role was to enroll data in accordance with a registration regulation that must be upheld by all businesses in the supply chain. Additionally, it is flexible enough to accommodate rescheduling or even outright cancellation. The inspection section monitors the subjects’ behavior and searches for signs of misconduct before rendering judgments and handing down penalties. Researchers were able to strengthen IoT access control and guarantee the safety of all permitted devices by keeping track of all important data and events in a distributed ledger. The loss of even a single node in a blockchain network might make reaching consensus unfeasible, leading to the eventual collapse of the network. The data of the peer with the problem might be lost if it does not commit immediately. By employing a backup peer, both the primary and backup peer may connect to the inspection server and start collecting real-time data. Without the requirement for a dedicated subchannel, sensitive information may be sent and stored utilizing the system’s built-in data segregation and communication modules. The projected method guaranteed the stability of the system by taking into consideration all aspects of network performance and scalability.

Considering aforementioned justifications, an immutable, transmittable identifier of authority should be used in “capability-based access control”. The identification serves as both a link to and authorization to utilize a certain resource. To gain access to a feature, a device must go through a different channel than the entity itself. Public capabilities may be accessed by any device, whereas private ones can be used by only those who have been granted access. The IoT node can “borrow” the capability it needs to get a storage pointer if it has previously produced and obtained it. Consequently, the form that a capability can take upon acquisition is determined at the moment of its creation. Bouras et al. [117] presented IoT-CCAC, an IoT consortium-specific distributed capability-based access control framework. Since it has the best qualities of both blockchain and traditional libraries, a blockchain-based repository is used to achieve excellent efficiency. The authorization process confirms (a) the validity of the token, (b) the approval of access right, (c) the availability of the asset, and (d) the fulfillment of conditions. The IoT-CCAC methodology performed satisfactorily and is well suited for smart city and enterprise network use cases.

By tracing the paths used by credentials, Li et al. [118] proposed “traceable capability-based access control (TCAC)” that makes it possible to revoke or modify access permissions. To facilitate the verification, denial, and modification of permissions, authors designed a novel competence token and established a temporal capability tree (TCT) that can produce privilege trends programmatically. Based on the results of the tests, TCAC is significantly quicker than both comparative methods, CapBAC [128,129] and xDBAuth [130], when it comes to confirming tokens and cancelling or updating them. A total of 73.3% of users/devices who were previously unavailable may now be located using TCAC’s assignment and random access features. This provides new insights into the relationships between permissions and delegation and opens up potential strategies for protecting IoT infrastructure throughout the use case environment.

Fossen [119] advocated a capability-based, four-part approach. When interacting with a RESTful API, the web app must save and utilize the tokens granted to the user by the “authority-server (AS)”. The AS tracks and controls the tokens and functionality. The RESTful API’s filter component does the verification and then passes the feature on to the API’s main logic. Logic handles both database connectivity and information processing. In a total of nine steps, each part interacts with all the others. The filter and the logic were the two main components of the RESTful API’s design; the filter was responsible for receiving HTTP requests containing access tokens, validating those tokens, and retrieving the corresponding attributes before passing them on to the logic. After receiving a request from a user, the RESTful API logic runs the requested protocol function for which the authority has granted permission. The implemented experiment ensured granular control over which API calls were made while using the RESTful interface. The framework enabled the programmer to define the user interface options for the system. Coarse-grained access control was employed in the traditional library implementation, with access representing an entity rather than an attribute of content. Unfortunately, a few important design choices (such as, proof-of-concept (PoC), delete-all, update-all, read-all, etc. and policy sheets for ruleset API) were ignored during access control implementation that restricts supporting complex data models.

One alternative is to implement “rule-based access control” (RBAC) [131]. Using RBAC, admins can grant or revoke privileges to devices based on their user profiles and the tasks they need to do. It is possible to categorize devices according to their responsibilities, since the amount of system access they need is often determined by the tasks they do inside a network. Roles that need access to the devices are then granted authorization to do so. If appropriate permissions are established for each task, access control will be easy to implement and maintain. The procedures of rule-based access control are as follows: the authorization rules are implemented throughout the whole access control system and are established by the security administrator. After the device has shown its legitimacy, its access is granted or denied depending on the comparison between the device’s permissions and the group policy. As a result of RBAC, the administrator can save time on procedural and ICT (information communication technology) support tasks, make the IoT system more secure and compliant, give each user and device only the permissions they need to do their job effectively, and free up extra throughput.

Saha et al. [120] advocated distributed Decentralized Hybrid Access Control for smart contracts (DHACS) for the IIoT (industrial Internet of Things). The idea of DHACS was to provide a more accessible, dependable, and secure access control mechanism for IIoT. The approach depends on the useful properties of blockchain technology, particularly the supply of smart contracts that makes possible a decentralized heterogeneous access control mechanism. The DHACS consensus process integrated many models for access control into a single solution, including role-based models, rule-based models, and organizational models. With the current set of ongoing operations and their related access constraints in mind, the activity pooler and block maker churned out blocks. While DHACS was initially developed for use in a private blockchain, it can be simply converted to function with a public blockchain or consortium blockchain to accommodate geographically scattered dependencies. For blockchain-based permission to be deemed completely decentralized, several entities must take part in the process of specifying and evaluating security regulations. There are a few challenges with the DHACS, such as how can a decentralized and lightweight access control solution be offered for distributed and enhanced IoT settings, given that IoT devices have limited computing and storage capacity? How can we ensure proper authentication (federated, completely decentralized, etc.)? Can an established framework be used, or do many configurations need to be combined? What criteria must be met when picking a blockchain scheme (private or public)? Attempting to combine RBAC with blockchain technology presented its own set of difficulties [132]. The first concern is the incompatibility (lack of standardization) of blockchain applications in various contexts and sectors. The incompatibility between RBAC and blockchain technology can be addressed by designing smart contracts that incorporate RBAC policies. The smart contract can specify access control rules based on roles and permissions, and these rules can be enforced by the blockchain network. The RBAC policies can be defined by IoT network consortium that is deploying the blockchain network, and the smart contract can be programmed to ensure that only authorized entities can access the relevant resources.

The second problem pertains to the amount of power consumed by the consensus mechanism that prevents wastage of resources. The process of reaching consensus can be energy-intensive, especially when using proof-of-work consensus algorithms. The third challenge relates to the latency that can be introduced by the additional bandwidth requirements of the blockchain network. Finally, implementing blockchain protocols in practical, nonindustrial environments presents a significant challenge.

Ubiquitous security can be achieved in a specific IoT context by enforcing security regulations, closed-loop monitoring, simplifying governance, and implementing comprehensive defense. PerBAC (pervasive-based access control model) was based on a strategy presented by El Bouanani et al. [121] that was developed after extensive research was conducted on several well-known access controls. This strategy was being distinguished by its interpretation of the decision-making algorithm, its depiction of abstract entities with features as a core principle, and the collaboration aspects necessary to maintain the case under a wide range of network conditions. When this algorithm is paired with information gathered from IoT environments, it could help improve access control decisions based on dynamic rules and entities. The proposed comprehension of the features, the dynamic entities, and their exploitation through the “aco” protocol generates a unique access control model tailored for the IoT paradigm. Thus, as per aforementioned context, in order to establish a safe connection between a client device and a server across a network, the server must first broadcast an identity to the client device. The service can comply with the user’s request if the identification is entered when the user is physically close to the service. Pervasive secure access is distinguished by its ability to detect risk at each point of interconnection using a variety of techniques (such as spotting anomalies in client behavior or considering contextual clues such as location and device, etc.) and to request enhanced authentication from the client only when necessary.

Yu et al. [125] proposed an Internet of Things (IoT) supply chain “secure data sharing scheme (SDSM)” that integrates blockchain technology with ciphertext-based entity encoding. This method can be used to set up tiered partnerships with different levels of access permissions. In addition, scholars proposed a metric based on the blockchain’s history ledger that could be used to rapidly establish the extent of relationships among users. To support partnership-based access controls, the approach combines participant-specific features into the ciphertext-based attribute cryptosystem. As a result, access permissions can be fine-tuned to a greater degree.

It is expected that, by 2025, edge devices such as smartphones, wearables, connected automobiles, and so on would be responsible for creating 10% of all content and processing 45% of all data [133]. Fog computing is expected to replace artificial intelligence, Internet of Things application development, and fifth-generation wireless networks (5G) within the next five years. It is an extremely virtualized system that connects user devices to traditional cloud data centers for storage and processing power. Fog computing is characterized by its low latency, position tracking, edge location, extensibility, real-time data and cloud interface, and online interaction support. To prevent modifications to cache and increase its reliability, Wang et al. [123] proposed a new lightweight label-based access control system (LACS). In order to ensure security, LACS checks the legitimacy of the approved fog nodes. Specifically, the LACS might verify the fog nodes’ eligibility for access to the caching service by verifying the authenticity of the shareable files containing encoded label values. Increasing efficiency and minimizing risks were the driving forces for the creation of LACS. The proposed technique successfully removed the noise source (the malicious fog node), freeing up some additional space for data storage, network bandwidth, and computational resources. However, its execution time must be reduced to the lowest possible measurable value (in milliseconds). On top of that, it verifies that requests are coming from approved fog nodes before denying them.

In fog-enhanced IoT systems, Zhang et al. [127] presented an outsourced access control scheme with hidden access structures (OAC-HAS). Three distinct benefits result from the OAC-HAS approach. In the first place, it presents a fog-cloud computing (FCC) setting that could be used for outsourcing. Then, it creates a method for external verification to ensure the integrity of data encryption activities carried out by fog nodes. Finally, it ensures confidentiality by sealing off access points to sensitive data. The suggested OAC-HAS method accomplishes a dynamic access policy, protects users’ privacy, and provides better precision in fog-enhanced IoT systems, as evidenced by the security evaluation and experimental outcomes.

In order to share information with gateways, applications, servers, and cloud infrastructures, IoT devices require reliable connections to the aforementioned nodes. Communication between devices and the cloud must be continuous and two-way for there to be an IoT. Remember that a good proof-of-concept may fail when deployed globally; therefore, make connectivity a major priority early in your Internet of Things project to avoid problems later on. The majority of internet connections today originate from cellular devices. Since it leverages already-existent global networks, cellular IoT is an obvious choice for many applications. Since cellular networks are already present in virtually every region of the world, the IoT was able to leverage this preexisting infrastructure. Lee et al. [124] introduced a density-clustering based “base station (BS)” modulation solution for lowering the power requirements of IoT networks (DeCoNet). To determine the optimal number of BSs and their optimal locations while accounting for differences in user density, experts used features gathered via density clustering methods to design the narrowing perimeter used to change the status of BSs in complete cellular IoT networks. Researchers determined the boundaries of each cluster’s area by averaging the distance between the cluster’s farthest border users in DBSCAN with the average routing in OPTICS. System-wide security necessitates network level slicing, the protection of application-based functions both in distributed clouds and in edge computing interconnections. In mobile telecommunication networks, there are four main routing components: the radio access network, the core network, the transport network, and the linking channel. Different types of traffic—signaling, data, and management—are carried by different layers of the network architecture. Every “sector” in a network consists of these three components. With regards to cybersecurity, all three layers are open to various forms of attack. The three-dimensional world is vulnerable to several threats that are universal. The access control system of a mobile network is essential. If the security of the core network or the management systems is breached, it might put the entire mobile network’s services at danger. By applying suitable access control, reduction in data consumption and increased energy efficiency can be achieved.

Sivaselvan et al. [126] argued that the software deployment of authentication protocols in IoT is riddled with security flaws, rendering the built-in authentication method useless. However, several of the security threats that are common on the Internet of Things can compromise the existing authentication methods for IoT that are not based on firmware. In addition, there are gaps in context-awareness, accessibility, compatibility, and cybersecurity in the state-of-the-art methods to access control for the Internet of Things. Due to these constraints, a reliable authentication and authorization solution was required to protect the ever-increasing number of IoT devices. As a result, Sivaselvan presented SUACC-IoT, a secure and trustworthy universal authentication and access management solution for the Internet of Things. Capabilities served as the foundation for the planned system, with each capacity acting as a token that granted authorized organizations access to the network. The capability token is utilized in the proposed system to manage and approve usage of scarce IoT resources. Simple cryptographic building blocks such as symmetric key encryption/decryption, message authentication codes, and cryptographic hashes are all that are required to operate the system. It was shown that SUACC-IoT is safe from the types of attacks often used in the IoT, including those that can be executed in probabilistic polynomial time.

Conclusively, it is worth highlighting that multiple factors increase the difficulty of implementing authentication and authorization protocols in an IoT setting. That is because most devices are typically limited by their battery, memory, network speed, and computing power. The throughput of popular authentication protocols makes most conventional verification and permission approaches unfeasible to run on resource constrained IoT devices. Another issue is that devices are occasionally placed in locations where it would be challenging or impossible to implement physical security measures.

In addition to this, there is an extremely diverse selection of hardware and software stacks that must be taken into consideration. As a result, in comparison to more conventional computer settings, we see widespread use of a wide variety of devices interacting via a wide variety of standards and protocols.

### 5.1. Cryptographic Paradigm in IoT

To ensure that only authorized entities have access to data transmitted across IoT networks, cryptography can be used for authentication and encryption. When it comes to cryptography in the IoT, there are a few tried-and-true methods. Symmetric key encryption [134,135] is often used to secure confidential data because of its efficiency and speed. Another kind of encryption used in the IoT is public key encryption [136,137], which uses a pair of keys—a public one for encrypting and a private one for decrypting. Encrypting data with asymmetric keys takes more time, but the private key is never exposed, making this technique safer. Cryptography with elliptic curves [138,139], in addition to symmetric key algorithms, is used in the IoT. Digital signatures that can be used for authentication are generated using elliptic curve cryptography. Lastly, a process called hashing [140,141], in which the data is converted into a code of a set length, can be used to assure data integrity. This technique ensures that no data has been altered in transit.

To confine the research boundary, we have identified the following lemmas:

(a) An IoT device’s security measures must meet the standards set forth by the IoT device application.
  - An “always-on” interoperability method is preferable to the periodic activation and deactivation of communication networks when an IoT system application has to transfer data often.
  - Data from IoT devices should be gathered as efficiently as possible and compressed before being sent over the network. Coordinating several IoT Services delivered by a single communications module within a single IoT device application is essential for making efficient use of the network.
  - To avoid seeming as though they are all functioning unison, application software operating on IoT devices should utilize a random pattern to request network connection.
  - The data should be encrypted from edge to edge using the IoT system software.
  - Before beginning any data transfer, the IoT device application must verify that it can communicate with the IoT platform. The level of security provided by the encryption technique should be commensurate with the IoT Service.
(b) The IoT-based technology must follow all required communication specifications.
  - The application running on the IoT device should not have to constantly reestablish the modem’s network connection.
  - When an Internet of Things device framework does not require constant information exchange and therefore may function with some delay in its IoT Service.
(c) IoT infrastructure should follow the throughput, latency, and yield-related parameters.
  - The Internet of Things device application must include “temporal resynchronization” capability, both for local and distant connections.

In order to conduct an accurate comparison of the various Internet of Things devices regarding communication technology and interoperability, there are a few essential factors that need to be taken into consideration. These crucial aspects include the type of protocol that is now being utilized, the spectrum of the connectivity, the throughput, the reaction time, the power efficiency, and the dependability. Zigbee [7], Wi-Fi [8], Bluetooth [9], LoRaWAN [14], and Z-wave [142] are just some of the popular choices accessible when it comes to protocols. There are also a lot of other possibilities available.

It is essential to keep in mind that the kind of protocol and interface that is utilized will change depending on the particular application, as well as the devices that are being utilized. For instance, if two different devices need to communicate with one another across a considerable distance, then a wireless protocol such as LoRaWAN may be the ideal option. On the other hand, if two devices need to communicate with one another over a short distance and the amount of data being sent is minimal, then a wired protocol such as Serial Peripheral Interface (SPI) may be the superior option. As a result, cryptography is applied in Internet of Things (IoT) deployments to safeguard the communication that occurs between the various devices. As a result, cryptography is applied in Internet of Things (IoT) deployments to safeguard the communication that occurs between the various devices. Examples of typical cryptographic approaches that are utilized in Internet of Things (IoT) communication include asymmetric-key algorithms such as Elliptic Curve Cryptography (ECC) and hash functions such as the Secure Hash Algorithm. Both algorithms are used to hash data. Another illustration of this would be symmetric-key algorithms such as the Advanced Encryption Standard (AES) (SHA). Additionally, cryptography can be used to safeguard the identities of devices on a network and to guarantee that data does not become corrupted or altered while it is being transmitted from one point to another.

Figure 4 illustrates the organizational structure for the classification of cryptographic techniques used on the Internet of Things. There is a basic difference between symmetric and asymmetric encryption in that the former uses the same key for both encryption and decryption, while the latter uses two different keys—one public and one private. When compared to symmetric encryption, asymmetric encryption is more secure but slower and less efficient, because the private key is not shared.

#### 5.1.1. Symmetric Ciphering

##### Block Cipher Methods

Using a symmetric block cipher, information is encrypted and decrypted in chunks of a fixed size. Combining it with an authentication mechanism that uses a secret key to encode and decode the data adds an extra layer of security, such as Hash-based Message Authentication Code (HMAC) [143] or Cipher-based Message Authentication Code (CMAC) [144]. Block ciphers can be used in many different modes of operation, allowing for the creation of novel encryption systems. In the IoT, block cipher methods provide strong encryption for secure data transmission and are well-suited for low-power devices due to their efficiency. However, these methods can be vulnerable to attacks if the encryption key is compromised or if there are implementation errors, and they may introduce processing delays, which can be problematic for real-time applications.

##### Data Encryption Standard (DES)

IoT networks frequently use the symmetric key encryption method known as Data Encryption Standard (DES) [145] to safeguard sensitive data in transit. It is a tried-and-true method of keeping sensitive data safe, and it is still widely employed today. When encrypting data, DES employs the Feistel network and a 56-bit key. Due to the rapid advancement of technology, DES is no longer encouraged for use in new applications because of its weak key size in comparison to more modern algorithms.

A more secure alternative to the DES algorithm is Triple DES (3DES) [146]. When compared to the original DES algorithm, 3DES’s key benefit is the greater security it provides. Since it employs not just one but three 56-bit keys, 3DES is considerably harder to crack than DES, which only employs a single 56-bit key. In addition, assaults such as related-key attacks are harder to pull off against 3DES. Last, but not least, 3DES is compatible with DES, making the transition from DES to 3DES a simple one.

##### Advanced Encryption Standard (AES)

Alternatively, there are three different key lengths available when using an Advanced Encryption Standard (AES) [147] to encrypt data: 128 bits, 192 bits, and 256 bits. An AES key used in an IoT network must be securely maintained and periodically updated to prevent unauthorized access. Computational complexity is the fundamental downside of AES. Compared to other symmetric encryption methods such as DES, it is a sluggish algorithm. Additionally, AES encryption necessitates extra memory, which might be an issue in embedded systems with constrained resources [148].

##### TwoFish

An improved version of the TwoFish encryption algorithm was presented by Hsiao et al. [149], which used a systematic method of design in conjunction with a chaotic masking-based method of exchanging encrypted data. Data transmissions employing chaotic synchronization have recently been called into question owing to attacks by hackers who deny data across the public network. Therefore, the TwoFish encryption method was combined with a chaotic synchronization strategy to foil hacker attacks. By doing so, the encrypted communication is shielded in a more secure network for transferring information. During our assessment, we found that the enormous block size (i.e., block size of 128 bits, which can support a key size of 256 bits) and complicated key scheduling of the TwoFish algorithm make it unsuitable for hardware implementation. On top of that, as compared to other block ciphers, the algorithm’s slowness makes it less acceptable for use in contexts that necessitate rapid processing. In addition, unlike more common algorithms such as AES.

TwoFish is a highly secure encryption algorithm that uses a large block size and key size, which makes it resistant to brute force attacks. Due to the computationally intensive nature of this encryption algorithm, it may not be suitable for IoT devices with limited processing power and resources.

Nevertheless, TwoFish has not been the subject of much cryptographic examination because of its low adoption rate. It is possible that this might lead to yet-undetected security flaws.

**Figure 6:**
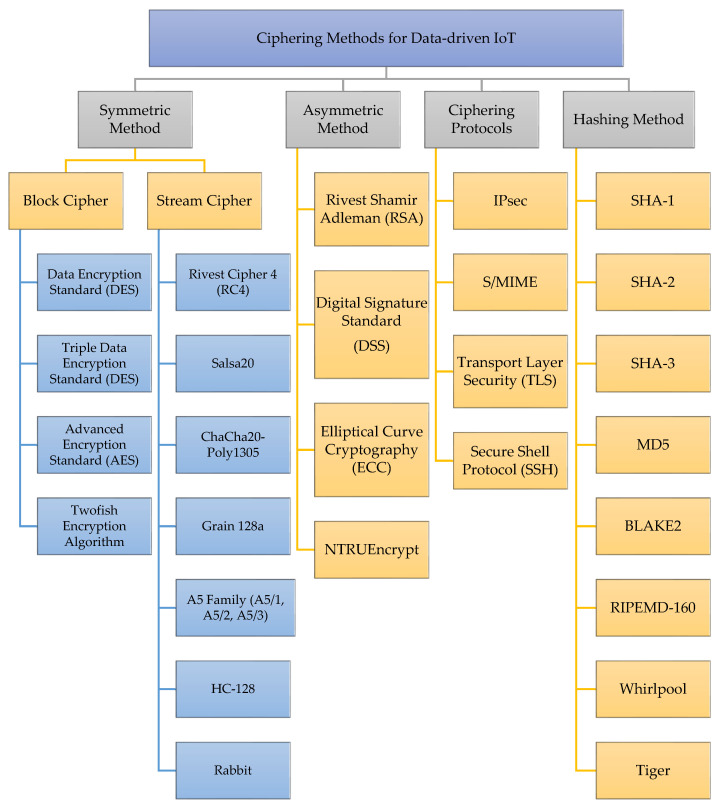
Taxonomy of cryptographic methods applied in the IoT environment.

Figure 6 presents a taxonomy of cryptographic methods applied in the IoT environment. This diagram systematically analyzes the security threats present in the IoT environment and the various cryptographic methods that can be employed to mitigate those risks. The taxonomy categorizes cryptographic methods into four primary groups: symmetric, asymmetric, ciphering protocols and hashing methods. Each group has several subcategories that describe the specific encryption methods that can be used in the IoT environment. Comprehensive details are furnished in subsequent sections.

##### Stream Cipher Methods

By using the exclusive-or (XOR) function, stream ciphers combine a stream of plaintext sequences with a stream of pseudorandom cryptographic hash sequences to encrypt data. If used properly, stream ciphers can provide a robust layer of security for a wide range of communications settings, including wireless networks, the Internet, and associated transactions [150]. In order to encrypt data, stream ciphers frequently use a keystream that is calculated from a secret key. Data bits or bytes are encrypted using the keystream, and then, the keystream is discarded.

1. RC4

The RC4 stream cipher [151] is now among the most widely used encryption tools because of its efficiency and simplicity of implementation. This stream cipher operates in bytes and allows for a wide range of key sizes. Depending on the use case, it can employ either a 64-bit or 128-bit key size. RC4 is a popular cipher; however, it has several security weaknesses that make it less secure than others, such as AES. Furthermore, a brute force attack against RC4 may be used to recreate the key if the attacker has enough time and the message digests. Given this, it is clear that alternative ciphers such as AES should be used wherever possible.

2. Salsa20

A 256-bit key payload encryption that does not preserve keystream blocks across transmissions is the specialty of the Salsa20 crypt family [152]. When set to counter mode, Salsa20 functions as a stream cipher that uses a checksum function to encrypt and decrypt 64-byte messages. This is why it is widely used in many encrypted contexts and considered safe.

Salsa20′s limited 64-bit nonce input hampers its usefulness in many applications. As a result of putting an emphasis on encoding efficiency rather than security, Salsa20 can become susceptible to certain attacks (i.e., side-channel attacks such as timing attacks) [153]. It can also be difficult to discern if decoding was successful, as there is no “crib” or known value in the plaintext. The lack of message integrity in Salsa20 presents an additional security risk that may reduce trustworthiness.

3. ChaCha20-Poly1305

The ChaCha20 [154] stream cipher generates a legitimate, seemingly random stream of bits by combining the outcomes of three basic arithmetic operations: addition, rotation, and exclusive-or (XOR). To guarantee the authenticity of a communication, Poly1305 authenticators generate two tags, an authentication tag and a verification tag. The ChaCha20-Poly1305 [155] method has a longer nonce intervals and was developed for collision-free random number generation. The ChaCha20-Poly1305 algorithm has certain problems because of its susceptibility to spoofing attacks. This means that a threat actor can create communications that seem to come from a wanted source while in reality they are falsified. The method is also susceptible to key reuse attacks, in which an adversary encrypts messages using a single nonce and decrypts them using a different key. Lastly, but not least, the method is vulnerable to collisions. As a result, an adversary who discovers two communications sharing the same authentication tag may be able to generate a third message using the same tag.

4. Grain 128a

Grain 128a [156] is a 128-bit key cipher that has an initialization vector (IV) that is 96 bits long and an output stream that is 128 bits long. A linear shift register, a nonlinear shift register, and an output function are the fundamental building blocks of the Grain-128a. Grain-128a is a lightweight cryptographic algorithm suitable for resource-constrained IoT devices.

As a result of its birthday paradox vulnerability [157], Grain-128a is an insecure system. This indicates that the cipher’s security could be breached if an adversary gained access to the pre-output stream and utilized it to find a distinguisher that could be used to divulge the key. Such an attack would impair the cipher’s ability to keep information private. As a result of its slow key setup time, Grain-128a is also unsuitable for applications that need speed. Since, Grain-128a has a maximum key size of 128 bits, which could be inadequate for certain applications.

5. A5 Family

The most widely recognized use for the A5 family of symmetric stream ciphers is as the cryptographic protocols used in mobile broadband and subsequent technologies. The A5 algorithms [158] are developed with a primary emphasis on both efficiency and safety, and they are meant to run on inexpensive, commodity servers. GSM is susceptible to attacks due to several shortcomings, one of which being the relatively short key length utilized in A5. The design and implementation of the algorithms are both incorrect, and both can be exploited by attackers, even those with limited computer capability. The decryption process might take place in a timeframe that is exceptionally near to real time.

(a) A5/1

A5/1 is utilized to construct a 114 input bits of output sequence for every spurt, and then that string is XORed with the 114 bits before the burst is modulated [159]. The initialization of A5/1 is accomplished by utilizing a 64-bit key in conjunction with a publicly available 22-bit subscript. The A5/1 cipher has a number of benefits, some of which include its relatively high resilience to well-known attacks, as well as its relatively minimal overhead, which makes it acceptable for usage on low-power devices. In addition to this, the key size is rather manageable, which contributes to its overall ease of implementation. The A5/1 cipher is vulnerable to linear and differential cryptanalysis, and its unchanging key requires regular key changes to stay secure. In addition, because of its minimal computing overhead, it is susceptible to cyber-attacks using brute force.

(b) A5/2

A weaker version A5/2 was proposed to offer a scalable solution for inter-GSM (Global System for Mobile Communications (GSM) network communication, allowing for backwards compatibility with older GSM hardware [160]. A5/2′s benefits lie in its minimal complexity and its capability to offer robust encryption with a small key size. As an added bonus, it has a significant degree of protection against common plaintext vulnerabilities. However, A5/2 has several drawbacks, such as being vulnerable to statistical attacks and not being capable of providing reliable encryption with larger key lengths. The key used to encode a particular message can be determined by an intruder using a related key attack, which A5/2 is vulnerable to.

(c) A5/3

The Kasumi algorithm [161], created by Mitsubishi Electric, is a block cipher utilized in 3G and LTE (long-term evolution) cellular standards, and it forms the basis of the succeeding version A5/3 [158,162]. Security analysis has shown that A5/3, which employs a 128-bit key, is impervious to all currently known attacks. The encryption of A5/3 is more secure, it can withstand more known plaintext attacks, and it provides better performance in embedded applications. The intricacy of the system increases the effort required for both configuration and administration, which is a drawback. In addition, A5/3 is more computationally complex than A5/1, which makes it more power-hungry and, as a result, less appropriate for usage in portable electronic devices.

6. HC-128

Wireless communications, secure data storage, and secure access to content are just some of the many use cases for HC-128, which is built to withstand linear and differential cryptanalysis [163]. OpenSSL [164] and Botan [165] are two examples of cryptographic libraries that support it, and its specifications can be found in RFC 4503 [166]. The HC-128 stream cipher is a great option because of its high level of security and reliable implementation. In addition to being extremely quick, it also requires relatively little memory. Key sizes can be anything from 64 bits up to 128 bits, providing an extra layer of protection.

Unfortunately, the requirement for a reasonably large array of 4 kb is the most significant shortcoming of the HC-128 method. This can be problematic for use when memory is constrained. The method is novel and not as frequently used as other algorithms, therefore interoperability concerns across applications are possible.

7. Rabbit

It is quite safe, since it uses 128-bit keys and a 64-bit initialization vector [167]. Compared to other popular encryption algorithms, Rabbit stands out due to its exceptional software efficiency, with a stated encryption/decryption throughput of 3.7 clock cycles per byte on a Core i7 CPU. It is effective in both software and hardware setups. Compared to other symmetric encryption techniques, Rabbit is more secure because it uses more complex functions for key formation and configuration transitions. These characteristics make it harder for an attacker to guess the cipher’s output. Furthermore, Rabbit is resistant to timing attacks since it encrypts each byte of data in the same amount of time. One limitation that Rabbit cipher may exhibit is that it uses a linear feedback shift register, which is not as secure as other applied algorithms [168]. Moreover, it has a limited key length of 128 bits, which makes it vulnerable to brute force attacks.

#### 5.1.2. Asymmetric Ciphering

Asymmetric Ciphering is used in the IoT for encryption and authentication. It offers scalability and flexibility for IoT applications, making it a great choice for secure communication. If there is a requirement for secure communication between two or more devices without the sharing of private keys, asymmetric encryption is the way to go. Its widespread use has led to its implementation in several practical contexts, including digital signatures and encrypted digital communication. Use cases for asymmetric encryption include remote access, file transmission, and the exchange of sensitive information. Furthermore, authentication and permission for safe access to networks and services are commonly provided using asymmetric encryption.

##### Rivest–Shamir–Adleman (RSA) Crypto Algorithm

RSA is a public key encryption method used in encryption for IoT technologies [169]. The RSA method is widely utilized in IoT applications, including SSL/TLS certificates, data encryption, and secure communication because of its reputation as an efficient and safe algorithm. The RSA method depends on how challenging it is to factor a huge integer into its prime components. The method employs both a public and private key for encoding and decoding. During our analysis and investigation, we have found that if the communication entity loses the private key, it becomes irreparable and may lead to security compromise. Furthermore, an adversary can read all communications if it controls the private key.

The size of the payload of an RSA-encrypted communication varies with the length of the message and the size of the RSA key. The typical payload size for a 2048-bit key is about 256 bytes. Although RSA supports encryption keys of any length, the most common sizes are 1024, 2048, and 4096 bits. Encryption is more robust when using bigger key sizes.

The advantages of using RSA in IoT include strong security, widely accepted and implemented, and suitability for key management. However, its disadvantages include high computational requirements, slower processing speed, and vulnerability to certain attacks if not implemented correctly.

##### Digital Signature Standards (DSS)

In order to ensure that all data transmitted over the internet is secure and legitimate, IoT relies on the Digital Signature Standard (DSS) [170,171]. The Federal Information Processing Standard (FIPS) defines methods for digital signature generation and verification. By using a specific DSS, information can be protected from modification during transmission. It also serves as an authentication mechanism for safe access to network-based resources. The major shortcoming of DSS is that there are many different digital signature standards and most of them are incompatible with each other, which complicates the sharing of data streams.

Depending on the technique and the message length, the payload size of a DSS signature can range from very small to very large. The standard payload size is between 40 and 50 bytes. DSS encryption keys are normally 1024 bits in length; however, this length can be increased for certain uses. For even more protection, IoT devices can make use of encryption keys that are 2048 bits in length or more.

##### Elliptical Curve Cryptography (ECC)

Public key encryption using elliptic curves (also known as Elliptic Curve Cryptography or ECC) allows for the generation of cryptographic keys that are lower in size, more secure, and more quickly generated than other methods [172]. As an alternative to the RSA cryptographic technique, ECC is widely utilized in applications and data storage. Due to its low power consumption, short key size, and efficiency in encryption and decryption, ECC is well suited for usage in IoT devices. ECC is utilized in digital signatures, encrypted file transfers, encrypted emails, and secure network access. The ECC security relies on the difficult-to-solve elliptic curve discrete logarithm challenge.

The payload size of ECC is determined by the type of ECC algorithm that is being used in conjunction with the length of the encryption key. The payload of an encrypted message is proportional to the size of the encryption key. For instance, the payload size and required encryption key size for ECC NIST P-256 and ECC NIST P-384 are both 256 and 384 bits, respectively [173]. Regrettably, ECC necessitates more computing power and memory than alternative public key techniques. Moreover, compared to other public key methods, ECC is more susceptible to quantum computing attacks; hence, it is essential to use a key size that is high enough to offer the appropriate level of security.

Thus, ECC offers strong security with smaller key sizes, making it ideal for resource constrained IoT devices. However, its implementation requires more computational power than traditional cryptography, which can impact performance and increase power consumption.

##### NTRUEncrypt

The lattice-based encryption system NTRUEncrypt [174] can be used to offer post-quantum security for IoT devices. Its lightweight architecture makes it ideal for protecting devices with limited resources, such as those seen in IoT (Internet of Things) implementations in the linked home, industrial, and automotive sectors. Since it makes effective use of resources, NTRUEncrypt is also an appealing solution for delivering post-quantum security to IoT devices. This makes it an interesting alternative.

Depending on the message length and the NTRU key size, the NTRUEncrypt payload size will vary. The normal payload size for the APR2011_439 configuration [175] is roughly 54 bytes. Keys greater than 439 bits are supported by NTRUEncrypt, however this is the default length. There is a direct correlation between key size and encryption security; bigger keys are more robust.

The primary drawback of NTRUEncrypt is the potential for higher storage and transmission costs due to the size of its public keys. The size of the key used determines how secure it is, and it can be computationally costly.

#### 5.1.3. Ciphering Protocols

IoT ciphering techniques are applied to encrypt data in transit to the cloud or between IoT devices. Verification and encoding are often used together in these systems. Cryptographic protocols such as IPSec, S/MIME, TLS, and SSH are used to secure data and communications over the internet. IPSec is a suite of protocols used for authentication and encryption of IP packets, while S/MIME is used for secure data transmission. TLS is the successor to SSL and is used to secure web traffic using HTTPS. Finally, SSH is a secure shell protocol used for secure remote logins and file transfers.

##### Internet Protocol Security (IPSec)

Encapsulating Security Payload (ESP) and Authentication Header (AH) are two instances of the suite of protocols known collectively as Internet Protocol Security (IPSec) [176]. The AH protocol protects the validity and integrity of the IP packet, whereas the ESP protocol protects privacy and secrecy. Due to the fact that one of the most important goals of the Internet of Things is to ensure the safety of data while it is being transmitted between various devices and the cloud, IPSec has become increasingly popular.

IPv6, the Internet’s next-generation protocol, incorporates IPSec by default; nonetheless, IPSec is backwards compatible with IPv4. Additionally, IPSec is not just backwards compatible with protocols such as TCP/UDP but also with cryptosystems such as the RSA and Digital Signature Algorithm (DSA). Furthermore, it can send data across public networks safely, since it can tunnel past network address translation (NAT) and firewalls [177].

Our analysis found that IPSec’s configuration and management can be challenging, making it one of the protocol’s key downsides. Moreover, IPSec requires two distinct protocols—the AH protocol and the ESP protocol—both of which can be challenging to maintain with and debug. In addition to that, IPSec is not equipped to deal with multicast traffic; thus, it could not work for your needs. Conversely, IPSec does not guarantee the confidentiality of transmitted data, since it does not provide end-to-end encryption.

##### Secure/Multipurpose Internet Mail Extensions (S/MIME)

The Internet of Things uses the encryption standard S/MIME (Secure/Multipurpose Internet Mail Extensions) while transferring data across the network [178]. In order to perform its two main functions, S/MIME requires the use of digital certificates for verification and encryption. When it comes to protecting the data provided by Internet of Things devices, enterprises can rest easy knowing that S/MIME provides the highest degree of encryption available. Users may quickly and easily adopt this protocol to secure their Internet-connected devices, because S/MIME is compatible with Windows IoT.

S/MIME’s include authentication, nonrepudiation, data integrity, and encryption. S/MIME requires a user to have an X.509 digital certificate with both a public key and a private key in order to function. Encryption is performed with the public key, whereas digital signatures are generated with the private key. A communication can only be secure if it is encrypted using the recipient’s public key before being sent. Digital signatures require the sender to sign the communication using their own private key. For verification, the recipient will utilize the sender’s public key.

In addition, S/MIME supports secure file transfer protocols such as FTPS and SFTP, and it can also be used for secure data transmission utilizing Transport Layer Security (TLS) [179]. Lastly, S/MIME also serves to establish a secure connection between web servers. One of the limitations of S/MIME is that it is not always feasible to authenticate the sender’s identity, which might lead to security concerns with regard to the management of identities.

Thus, S/MIME is a suitable choice for IoT devices that require secure communication with end-to-end encryption and digital signatures to protect sensitive data. Nonetheless, the need for exchanging public keys can pose difficulties in deploying it on a large scale. Moreover, the intricate and resource-intensive implementation of S/MIME can have an impact on the performance of devices with limited resources.

##### Transport Layer Security (TLS)

To protect sensitive information and guarantee message authenticity, integrity, and nonrepudiation, TLS is utilized. TLS enables authentication using digital certificates and supports several different encryption techniques [180]. TLS also reinforces a number of key exchange protocols, such as RSA and Diffie-Hellman, in order to enable clients and servers to engage in secure connection handshakes with one another. TLS 1.3 is the most recent version of the protocol, and it incorporates a few extra security measures [181]. These additional security features include forbidding Rivest Cipher 4 (RC4) negotiation, perfect forward secrecy (PFS), and required message-digest-5 algorithm (MD5) cryptographic hashes.

The overhead that relates to the TLS protocol can be problematic for IoT servers that have a restricted amount of processing power and memory. This is one of the downsides of the TLS protocol. In addition, TLS has the potential to be susceptible to man-in-the-middle (MiTM) attacks [182]. However, TLS can also increase the processing overhead and communication latency, which may be problematic in resource-constrained IoT devices with limited processing power and battery life.

##### Secure Shell Protocol (SSH)

IoT makes use of a network protocol known as the secure shell protocol (SSH) to provide safe access to a variety of resources and systems. SSH is utilized in the process of securing, configuring, managing, maintaining, and operating network routers, servers, and other components of core network [183]. Authentication, data encryption, and data integrity are all covered by SSH protocols. An SSH connection encrypts data and communication between two devices. Data is encrypted with a symmetric key algorithm while using SSH, such as with AES or DES. In addition to this, it authenticates the user by employing an asymmetric key method, such as RSA, to exchange keys with them. When authenticating communications, SSH is also capable of using a variety of hashing algorithms, such as SHA-1 (Secure Hash Algorithm 1) and MD5. In addition, the SSH protocol supports a variety of tunneling protocols, such as port forwarding and X11 forwarding, which enables users to safely tunnel network connections by using the SSH protocol.

Since SSH does not provide authentication of the server, an IoT device could not be aware if a malicious server is being utilized, because SSH does not support authentication of the server. As SSH is not inherently compatible with a few operating systems (e.g., Tizen OS v4.0), it is possible that additional software may need to be installed in order to use it [184]. This is another one of SSH’s many downsides. In fact, SSH is known to make extensive use of a server’s resources, which might inhibit its overall performance. In addition, SSH calls for the use of an external authentication system and calls for the environment to be appropriately set up for security.

#### 5.1.4. Hashing (Integrity)

IoT apps employ hashing algorithms such as MD5 and SHA-1 to keep data safe. Hash methods are used to generate a fixed-length outcome from an input of arbitrary length. The hash has the potential to detect any modifications to the primary data, making the latter unchangeable and secure [185].

SHA-1 and SHA-2 are the two most common secure hashing algorithms. SHA-1 is a relatively ambivalent algorithm that generates a 160-bit hash, while SHA-2 is more secure and generates a 256-bit hash. The most up-to-date member of the family, SHA-3, generates a hash value of 512 bits [186].

The fundamental benefit of adopting hashing algorithms in Internet of Things applications is that they provide a safe method of authenticating data, ensuring that the data has not been tampered with and is originating from a trustworthy source [187]. Hashing algorithms are perfect for use in encryption, since they are quick to compute and produce a different hash for each input.

##### SHA-1

SHA-1 creates a 160-bit hash, considerably boosting the security and integrity of stored data. In order to verify that information has not been tampered with in transit, the SHA-1 technique is used to generate a unique hash for each input. SHA-1 is widely used in IoT applications since it is fast and can generate a different hash for each input, and it is also significantly faster than its counterparts [188]. To verify that a message has not been tampered with, another technique called HMAC (Hash-based Message Authentication Code) is used, and it makes use of SHA-1.

The primary drawback of SHA-1 is that it lacks security; as a result, it is now deemed obsolete and is no longer recommended for use in applications that need high levels of security.

##### SHA-2

Numerous IoT implementations rely on the secure hashing technique SHA-2 for data transmission and storage. It is a hash function used in cryptography that generates an output of a predetermined length from the input. When compared to its predecessor, the Secure Hash Algorithm 1 (SHA-1), SHA-2 offers increased security and resistance to common attack vectors [189].

To generate a fixed-length hash from an input message of arbitrary length, SHA-2 relies on the Merkle-Damgard technique. It is made up of four individual algorithms: SHA-224, SHA-256, SHA-384, and SHA-512 [190]. The hashing algorithms SHA-224 and SHA-256 use 32-bit blocks, whereas SHA-384 and SHA-512 employ 64-bit blocks.

SHA-2′s key benefits are its security, speed, and reliability. The security provided by SHA-2 is far superior to its predecessor, SHA-1. In addition, it is quicker than SHA-1, which makes it applicable for a far wider variety of applications. As an added bonus, SHA-2 is secure, since it can withstand lengthier attacks [191].

The most significant disadvantage of SHA-2 is its compatibility, since the length of the hash, which is 256 bits, may be excessively large for certain applications.

##### SHA-3

Internet of Things applications use the cryptographically robust hashing algorithm SHA-3 to hash data securely. Using SHA-3′s hashes is a reliable way to ensure the safety and privacy of data with a 512-bit size [152]. SHA-3 is a reliable hashing algorithm, since it cannot be compromised by collision attacks [192], unlike SHA-1 and SHA-2. Furthermore, SHA-3 is superior to other algorithms in speed and its ability to generate a different hash for each input, making it a prime candidate for use in cryptography.

The main problem of SHA-3 is compatibility concerns. Incompatibilities can occur between applications and Runtime environments that do not support SHA-3. The 512-bit hash length may also be excessive for some applications.

##### MD5

IoT applications frequently use MD5 (message-digest-5), a cryptographic hash function, for purposes including data error checking, authentication, and digital signatures. It is designed to have minimal collisions and has a low computational cost [193]. Therefore, it can be implemented in systems that need a high level of safety.

MD5′s key benefits in IoT applications are its efficiency, low collision rate [194], and strong security. It is very simple to use and incorporate into programs, because it is widely supported in most programming languages.

The MD5 hashing algorithm is susceptible to collisions, which is the primary drawback of employing it in IoT applications. The likelihood of two distinct inputs yielding the same hash is minimal, yet it is nevertheless possible. This could lead to problems such as authentication bypass.

##### BLAKE2

The BLAKE2 hash function is significantly quicker than Sha-256, while yet being at least as safe as the accepted standard, SHA-3. For the optimum efficiency on multiprocessor or parallel processing CPUs, BLAKE2 provides the eight-way parallel BLAKE2sp, as well as the four-way parallel BLAKE2bp. When it comes to the Internet of Things, BLAKE2 has the algorithms that are needed to meet network specifications. BLAKE2 is at its best on 64-bit processors, where it can achieve throughputs of one gigabyte per second (3.08 cycles per byte) on an Intel Core i5-6600 [195].

The BLACK2 hash’s primary benefits are its high resilience to brute force attacks and its low memory requirements. In addition, the technique has modest computing requirements, making it suitable for use in low-power Internet of Things devices. The main disadvantage of the BLACK2 hash is that it is vulnerable to collision attacks [196].

##### RIPEMD-160

Internet of Things (IoT) applications make use of a cryptographic hash function called RIPEMD-160, which has a bit length of 160. The RIPEMD-160 algorithm produces a one-of-a-kind, unforgeable hash for each input, which can be used to check the validity of the information [197]. It is immune to collision attacks, and RIPEMD-160 is regarded more secure than its predecessor, RIPEMD-128. RIPEMD-160 is ideally suited for use in encryption, since it is quicker than many alternative algorithms, and it can provide a unique hash for each input.

The fundamental flaw of the RIPEMD-160 cryptographic hash algorithm is that its output is only 160 bits in size. This renders the system vulnerable to brute force attacks, since an attacker may try every possible value for the 160 bits until they discover one that produces the same result as the message being cracked.

##### Whirlpool

For the purposes of encryption and data integrity checking, Whirlpool generates a 512-bit hash. The Whirlpool algorithm creates a unique, immutable hash for each input, which can be used to determine whether data is legitimate. Since it is immune to collision attacks, Whirlpool is a more robust hashing algorithm than SHA-1, SHA-2, and RIPEMD-160 [198].

Whirlpool’s primary drawback is that it needs more memory than other hashing algorithms to maintain and analyze the data. Furthermore, length extension attacks may be used against Whirlpool, allowing an attacker to take a known hash and append additional characters to it without altering the resultant hash.

##### Tiger

The Tiger cryptographic hash method was developed specifically for remote systems. Any message submitted to the algorithm will be converted into a 128-bit digest. Tiger can withstand brute force attacks while yet being incredibly lightweight and functional [199]. Tiger’s key benefit is that it uses minimal processing power, making it suitable for use in battery-operated Internet of Things gadgets.

Tiger hashing is vulnerable to pseudo-near-collision attacks, which can be used to create two different inputs that produce the same hash.

## 6. Roadmap for Securing IoT Devices

The exponential growth of IoT devices has rendered them vulnerable to cyber-attacks, necessitating the development of a roadmap to secure these devices through the formulation of guidelines and standards. Such a roadmap must consider the challenges inherent in securing IoT devices, including the diverse range of devices and communication protocols, resource limitations, and the need for Denial-of-Service (DoS) resistance. Additionally, it must consider end-to-end security, novel network architectures, bootstrapping of a security domain, and operational challenges. The security measures implemented must be able to handle the complexities associated with mobility and IP network dynamics, ensure cryptographic agility, and safeguard individual privacy. Furthermore, incorporating Artificial Intelligence (AI) and Machine Learning (ML) to enhance IoT security is critical. By leveraging AI and ML, entities can significantly improve their ability to safeguard sensitive data, forestall cyber-attacks, and minimize the risk of business disruption.

Likewise, the quantum security rules related to IoT can have a significant impact on the security of IoT devices and networks. Quantum computing has the potential to break many of the cryptographic algorithms that are currently used to secure IoT devices and networks. This means that sensitive data, such as personal and operational information, can be at risk of being accessed by unauthorized parties.

However, the development of quantum-resistant cryptographic algorithms can help mitigate these risks. These algorithms are designed to withstand attacks from both classical and quantum computers, making them a more secure option for IoT devices and networks.

In addition, the implementation of quantum key distribution (QKD) can provide a higher level of security for data transmission between IoT devices. QKD is a method of encrypting data using quantum principles, which can make it impossible for a third party to intercept or access the data without being detected.

In context of access control paradigm, one promising future direction is to incorporate contextual information into decisions. This means considering the node’s location, time of day, and other factors when determining whether to grant access to a resource. Another direction is to leverage the power of blockchain technology to implement decentralized access control mechanisms that can provide better security and privacy guarantees. Ultimately, access control policies need to be updated and refined continually as new threats and vulnerabilities emerge.

### 6.1. How Can Artificial Intelligence (AI) and Machine Learning (ML) Change IoT Security?

As technology advances, so do the threats to security posed by malicious actors. Artificial intelligence (AI) and machine learning are two technologies that can be used to protect IoT systems from cyber-attacks in the future.

Fundamentally, AI can be used to analyze massive amounts of data quickly for potential anomalies or suspicious activities, which could indicate a breach in security protocols. By leveraging powerful algorithms, AI can detect patterns faster than humans ever could and alert network administrators when it finds something out of place before any damage is done. This type of proactive approach will help ensure that networks remain secure even if attackers try novel methods or techniques not seen before by traditional cybersecurity solutions, such as firewalls or antivirus software programs.

Subsequently, machine learning offers another layer of protection against cyber-attacks because it allows computers to learn from past experiences without being explicitly programmed with rules on how they should react in certain situations. Machine learning models also allow for real time analysis meaning they are always up-to date on current threats while still being able to identify new ones as well; this helps reduce false positives caused by outdated threat signatures found within legacy systems, making them more reliable overall compared to other existing solutions out there today.

Ultimately, both AI and machine learning offer scalability, which means these technologies will not require additional resources every time you need them due to their ability to self-adjust according to different parameters set forth beforehand. With all these advantages combined into one comprehensive package makes clear why many experts believe utilizing artificial intelligence and machine learning-based IoT security solutions will become standard going forward, protecting device relay data better than ever before.

Adversarial attacks, in which an adversary deliberately manipulates data to deceive the system, are a serious problem for AI and ML, and some algorithms are difficult to read, leading to challenges in identifying the underlying cause of security breaches [200]. Techniques such as explainable AI and adversarial training have been offered by researchers as ways to address these issues. Artificial intelligence and machine learning have their drawbacks, but they also have their benefits, such as the ability to process massive volumes of data and spot anomalous behavior in real time [201]. For this reason, including explainable AI and adversarial training can improve the trustworthiness and reliability of IoT systems by providing transparency and understanding of the AI models and their decision-making processes, as well as enhancing the resilience of these systems against potential cyber-attacks and adversarial behavior. This can ultimately lead to better performance and user experience and increased adoption and acceptance of IoT technologies.

### 6.2. Risk Factors

The development of AI and ML-based IoT security is a double-edged sword. On the one hand, it offers many advantages in terms of increased efficiency and improved protection from cyber threats (i.e., as stated in an earlier section); on the other hand, there are potential risks associated with its use that must be carefully considered. In this section we have discussed three key areas where AI and machine learning can pose a significant risk to IoT security: privacy concerns, algorithmic bias, and data breaches.

To begin, there are security and privacy concerns associated with IoT devices that use artificial intelligence. As these systems become increasingly sophisticated at recognizing patterns in user behavior or detecting anomalies in system usage data, they may inadvertently collect personal information without users’ knowledge or consent, potentially leading to serious violations of individual rights, such as the right to privacy under GDPR regulations. It is important for IoT network administrators to use these technologies to ensure that their policies protect against unauthorized collection while still allowing them access only to the necessary data needed for practical monitoring purposes.

The second issue is the prevalence of acknowledged bias in AI technology employed by businesses, from parametric search engine results to facial recognition software used by law enforcement organizations worldwide. The incorrect detection of hostile behavior on an IoT network can lead to social injustice and false alarms if the algorithms employed to make those judgments are biased. Before implementing new algorithm-based solutions, businesses should do thorough testing, independent audits, and frequent reevaluation procedures.

Furthermore, organizations should spend the time and effort to set up secure configurations across all connected devices. They should also ensure that the patching and updating process is carried out regularly to close any newly discovered vulnerabilities before they are exploited maliciously.

Ultimately, while there are numerous benefits given by the deployment of AI and ML-driven Internet of Things security systems, there are also substantial concerns that must be considered, lest we face tragic results in the future if appropriate precautions are not taken right now.

### 6.3. Future Directions: Decisive Remarks

We envision the following future directions of IoT security in the context of ML, AI, and pervasive computing:

(a) Development of lightweight and efficient ML and AI models for IoT devices with limited resources while ensuring their security and privacy.
(b) Exploration of new security models and protocols for securing communication and data exchange among heterogeneous IoT devices and networks.
(c) Adoption of pervasive hardware-based security solutions such as trusted execution environments, secure boot, and secure storage to strengthen the security of IoT devices and prevent physical tampering and attacks.
(d) Integration of adaptive and scalable blockchain technology to enable secure and transparent data sharing and management among IoT devices and stakeholders.
(e) Design and development of new authentication (e.g., enabled with lightweight quantum encryption) and access control mechanisms (e.g., continuous authentication, context awareness, decentralization, and user-centric) to ensure the integrity and confidentiality of IoT data and prevent unauthorized access and usage.
(f) Exploration of new models and frameworks for addressing privacy and ethical concerns in the collecting, processing, and sharing of IoT data, particularly in pervasive computing.

## 7. Conclusions and Our Recommendations

The research indicates that, as IoT technology advances, the associated security risks also increase. The increasing frequency of cyber-attacks involving the exploiting of a typical IoT device to gain access and compromise the entire network has underscored the importance of IoT security. Ensuring the safety of networks that rely on IoT devices has become an essential priority. IoT security encompasses a diverse range of techniques, strategies, protocols, and actions that aim to minimize the growing vulnerabilities posed by IoT to modern businesses. To ensure that the IoT networks are protected from potential attacks, it is crucial that computer science/engineering experts, such as researchers, scientists, and academics, stay up to date with the latest IoT security solutions.

This paper provides a comprehensive overview of the most critical security considerations related to the Internet of Things. We briefly review how literature suggests using IoT security on various tiers. Furthermore, we give a transient breakdown of the IoT threat vectors and mitigation strategies. We analyze the consequences of the attack and link them to the countermeasures that have been recommended.

IoT-enabled enterprises ought to understand and develop comprehensive monitoring tools to quickly detect any unusual activities on their networks. These tools should utilize advanced detection tools by exploiting enabling technologies, such as machine learning and artificial intelligence algorithms, to analyze large datasets generated during normal network operations and alert administrators of deviations from the norm. By allowing organizations to respond quickly to security incidents and anticipate potential threats before they occur, layered-focused defense mechanisms can significantly minimize the downtime resulting from cyber-attacks.

## Figures and Tables

**Table 1:** Assessment of wireless IoT technologies. ‘x’ means not-available, and ‘✓’ means available.

Wireless Communication Technology	ZigBee	BLE	6LoWPAN	LoRaWAN
**Device Interconnectivity**	Mesh topology	Mesh topology	Mesh topology	Star topology
**Spectrum**	1–100 m	Approximately 10 m	~200 m max.	6.2 miles max.
**Sleeping Mode**	12 μA	9 μA 0.4 μA at low energy	12 μA	7.66 μA to 34 μA
**Awake Mode**	50 mA	35 mA	>40 mA	>34 mA
**Transmitting Mode**	52 mA	37 mA	>50 mA	>52 mA
**Extensibility**	✓	x	✓	✓
**Intercommunication**	x	x	✓	✓
**Current Consumption** *(VBAT)*	3.3 V	3.3 V	3.3 V	4.2 V
**Application (examples)**	Home Automation, Embedded Sensing, Industrial Control Systems	Transfer Data Files, Data Logging Equipment, Short-range Data Transmission	Transmission of IPv6 Packets, Wireless Sensor Networks (WSN), Low-power Mesh networks	Smart City, Chirp Spread Spectrum (CSS) technology
**Digital Input Status**	1 or 0	1 or 0	1 or 0	1 or 0

**Table 2:** Threats to the security of the Internet of Things.

Attack	Portrayal	Purpose
Intrusion into a node [29,30]	IoT nodes are the components of an IoT ecosystem that facilitate data transfer between devices in the real world and the cloud. These devices are envisioned as aggregators of data from several sensors coming from various sources. The attacker makes changes to the nodes or disrupts their functions and then acquires full control of the node. This exploit damages the hardware, which will reduce the availability of resources.	Attacks that entail tampering with the device can take advantage of newly discovered vulnerabilities and exploit them. In point of fact, they are a type of physical attack in which the attacker attempts to break security after first attempting to corrupt the memory or the computation, and then gaining further knowledge by interacting with the IoT equipment. This occurs after the attacker has attempted to break security by corrupting the memory or the computation. Following this, the attacker will try to circumvent the security.
Node Dependency Injection [31,32]	When one or more dependents (or services) are “injected” into a reliant entity, they are given by reference to the instrument. This can be done with several dependents at the same time. The term “dependency injection” refers to this specific approach to creating software applications. Moreover, when an adversary injects fake nodes into a complete network, this is known as fake node injection. This occurs when the adversary inserts counterfeit nodes into the linked authorized nodes of the network.	It grants the attacker the ability to exert control over the data stream. The need to govern the data flow will arise as a direct consequence of this attack. Any kind of data might be susceptible to being processed by an opponent, who could then take control of it. A variety of different physical devices might be compromised by using this attack. When working in an environment such as this one, establishing reliable connections between every sensor/IoT node and the user who will ultimately be using the system is of the highest importance.
Node Acquisition Attack [33,34]	A typical example of a physical attack on a network is the capture of a node and subsequent extraction of its ciphering data. Later, it will be used to initiate more exploits within the network. There are several different types of IoT Node Acquisition Attacks, including spoofing, jamming, physical compromise, supply chain attack, firmware attack, and malware.	The adversary’s purpose is to trick the unidentified nodes into thinking they are at a known location by manipulating the signals between known nodes or by posing as a recognized node to falsify, change, or replay signals. With that in mind, precautions must be taken to ensure the estimated locations remain accurate even when under attack.
Eavesdropping Attack [35,36]	Eavesdropping attacks, in which hackers listen in on normally private network traffic, can successfully target any device connected to the Internet of Things (IoT). Types of eavesdropping attacks include passive eavesdropping, active eavesdropping, phishing and spear phishing attacks, drive-by attack, password attack, SQL injection attack, cross-site scripting (XSS) attack, eavesdropping attack, device fingerprinting, malware, and AI-powered IoT-based attacks.	Eavesdropping can occur when a connection between two endpoints is neither secure nor sufficiently strong. This leaves the link vulnerable to assault. Lack of encryption, outdated software or hardware, malware infection, or a mix of the three is all potential causes of unprotected switches and routers. Eavesdropping attacks might yield a large reward for the perpetrators. Information such as credit card numbers, names, addresses, phone numbers, email addresses, passwords, and even privileged information might be stolen.
Node Cloning [37,38]	Most IoT installations are placed in unrestricted, potentially dangerous areas. Consequently, they are extremely susceptible to intrusions through clone nodes or node replication. Some common types of Node Cloning Attacks in IoT are physical cloning, software cloning, address spoofing, configuration injection, replay attack, and side-channel attacks.	Capturing valid IoT devices allows the attacker to extract them, get access to important data such as node ID and keys, and launch a replication operation. If an attacker uses this vulnerability, he or she can eventually take over the entire network and do any action permitted by the approved nodes.
Radio Frequency (RF) Jamming [39,40]	One of the most common and successful forms of exploitation is the jamming of infrastructure communication, which disrupts or halts data transmission through the system. Jamming occurs when an adversary takes over the channel where legitimate nodes are trying to communicate. Many methods exist for an attacker to cause interference and clutter in the wireless frequency. One method is to flood the network with noise in the form of continual, uncontrolled data transmissions. It is also possible that there is a constantly high volume of data transmitting valid frames but just taking up all the available bandwidth. Some of the common types of RF Jamming attacks include pulse jamming, continuous wave jamming, random jamming, selective jamming, and reactive jamming.	The majority of devices include RF capabilities for sharing data wirelessly. A denial-of-service attack can be caused by any circumstance preventing normal data transmission.
Replay Attack [41,42,43]	When a malicious user falsely delays or retransmits a secure network message to trick the intended recipient into performing the hacker’s desired action, this is known as a replay attack. Several types of replay attacks can be used against IoT devices, including authentication replay attacks, session hijacking replay attacks, and encrypted data replay attacks. In an authentication replay attack, an attacker can intercept an authentication request and then delay or repeat it in order to gain access to a system. In a session hijacking replay attack, an attacker can intercept a valid session and use it to bypass authentication and access the system. In an encrypted data replay attack, an attacker can intercept and replay encrypted data in order to gain access to confidential information.	To steal private information, hack into secure networks, or make identical transactions are common goals of replay attacks. To protect against these types of attacks, devices should be secured with encryption, authentication, and authorization. Additionally, monitoring devices for suspicious activity and regularly patching any vulnerabilities is important.
Device Spoofing Attack [44,45,46]	Spoofing occurs when a linked IoT network suffers a cybersecurity failure at a lower layer. When, for instance, a computer system containing financial information is connected to the same IoT network as a Zigbee-enabled smart appliance with minimal security measures in place. In this context, “device spoofing” refers to the process of impersonating a different device using specialized software. The tools can fake software and hardware characteristics to deceive surveillance software. Some of the most occurring vulnerabilities are MAC address spoofing, IP address spoofing, DNS spoofing, HTTP spoofing, IoT cloud spoofing, and node spoofing.	It is a practice of impersonating a trusted entity to gain access to protected resources, commit fraud, steal sensitive information, obtain financial gain, or distribute malicious codes. A wide variety of spoofing attacks relate to data, domain, IP, and ARP. A few methods can be used to help prevent an IoT device spoofing attack. The first is to ensure that all devices on the network have strong authentication methods in place, such as passwords and two-factor authentication. Additionally, it is important to keep all devices up to date with the latest security patches and updates. It is also important to use encryption technologies such as a Secure Socket Layer (SSL) or Transport Layer Security (TLS) to protect data transmissions. Finally, it is important to enabling firewalls and network address translation (NAT) to protect the network from outside access.
Sleep Deprivation Attack [47,48,49]	As part of a sleep deprivation or denial-of-sleep exploit, a malevolent device transmits requests to target endpoints only at the frequency required for keeping them active. Therefore, unlike in a barrage assault, the target devices are kept awake but are not forced to carry out any particularly taxing tasks. There are several types of IoT sleep deprivation attacks. These include flooding the target device with requests, sending malicious code to disrupt its sleep cycle, and exploiting vulnerabilities in the device’s firmware to prevent it from entering a low-power state. Additionally, some attackers may attempt to use social engineering tactics to induce the user to keep the device active and thus prevent it from entering a low-power state.	The interactions are meant to prevent the victim node from entering a power-saving sleep state. As a result, the victim’s lifespan can be drastically shortened by this attack.
Temporary Disabling Attack (TDA) [50,51]	The malware injected into an IoT system by an attacker can compromise its integrity and allow for the theft of sensitive information or the implementation of additional attacks. Furthermore, if vendors do not guarantee proper software protection, certain systems might be compromised with viruses’ right out of the box. IoT devices are particularly vulnerable to TDA attacks because of their limited processing capability. Thus, several types of IoT temporary disabling attacks exist, including exploiting hardware or firmware vulnerabilities, distributed denial-of-service (DDoS) attacks, flooding servers with requests, and using malicious scripts to disable devices. Additionally, attackers may use physical access to the device to temporarily disable it, such as unplugging it or removing its battery.	Denial-of-service attacks, such as TDA hinder a system’s ability to handle legitimate requests by flooding it with spam. In most cases, these attacks are used to disrupt services or interfere with the normal functioning of the device, but in some cases, the goal may be access to the device or its data.
Unauthorized Access Attack [52,53,54,55]	The term “unauthorized access” describes when hackers enter a system without authorization. Passwords that are too easy to crack, a failure to safeguard against thought control, hacked credentials, and even insider threats all contribute to the prevalence of these types of attacks. Types of unauthorized access attacks include a brute force attack, man-in-the-middle attack, IoT-focused phishing attacks, and social engineering attacks that attempt to trick device users into revealing sensitive information. Extensive monitoring of shadow devices and regular software updates can furnish safeguard against such attacks.	Access is gained to an IoT system by an unauthorized user/device who intends to commit an attack of some kind, whether it relates to data theft, system destruction, or the activation of a ransomware vulnerability.
Authentication and Authorization [56,57,58]	Whether an individual is using an IoT device for home automation or a major corporation is using hundreds of IoT devices to track and monitor processes and resources, authentication and authorization are crucial components of security breaches. The first step in developing an IoT authentication and authorization strategy is gaining an in-depth knowledge of the organization’s IoT usage and network communication patterns. Some common types of authentication and authorization attacks in IoT include password attacks (i.e., brute force or dictionary attacks), man-in-the-middle attacks, spoofing attacks, repudiation attacks, and session hijacking attacks.	For devices that can only communicate to one other device, “one-way authentication” is the best option for establishing trust. It is still important to implement security measures, although constant monitoring is not required for such setups. The process of two devices engaging with each other verifying the identities of the other device before transferring data is referred to as “mutual authentication”. Both devices need to be able to compare their IDs and maintain access to the technologies used by the other gadgets. Unless both devices trust each other’s digital certificates, there will be no way for them to talk to each other. Through the TLS protocol, certifications can be transferred and compared. Devices are authorized in three separate ways using the “centralized three-way authentication” approach, which requires a valid digital certificate to be registered with a central authority or server. The trustworthy third-party acts as an intermediary between the communication devices to facilitate the transfer of cryptographic guarantees. Hackers are unable to steal the three-factor authentication security certificates since they are not stored on the devices.
Buffer-overflow Attack [59,60,61]	Overflow happens when more data is being stored than can fit in a buffer. When the IoT application tries to save the input to the buffer, it writes over the neighboring system memory, which can cause serious issues. A system can be exploited by malicious actors that are aware of its storage architecture if they purposefully feed it data that exceeds the capacity of the buffer or if they get access to the system and modify the source code stored in the system’s memory. Buffer overflow attacks in IoT can take several forms, including stack-based buffer overflows, heap-based buffer overflows, format string attacks, integer overflows, and stack smashing.	By far, the most common kind of buffer overflow exploits the ephemeral data on the stack, which is reserved for use within a function. It is more challenging to carry out a heap-based attack since doing so necessitates using more memory than the system has allotted to the program, which is needed for its continuous dynamic activities. To prevent this type of attack, IoT developers should ensure that their code is secure by using secure coding practices and ensuring all buffers are properly allocated and all input is validated. Additionally, security patches should be consistently applied to ensure that any newly discovered vulnerabilities are addressed.
DDoS (Slowloris) Attack [62,63,64]	Slowloris is an application layer distributed denial-of-service (DDoS) attack that overloads and eventually shuts down a target Web server by repeatedly delivering unclear Hypertext Transfer Protocol (HTTP) requests from a single IoT device. This specific DDoS attack can be launched with minimal bandwidth while leaving other applications and ports unaffected. A Slowloris attack can take several forms, including HTTP flood, SYN flood, DNS amplification, SQLi, ICMP Echo request attack, and fraggal attack.	The attack of a Slowloris is deliberate and meticulous in nature. A series of incomplete connection requests are sent to the vulnerable central server. Therefore, the intended server responds to requests by opening additional connections. All the server’s available sockets for connections will be used up quickly, preventing any new connections from being established. In the end, even though it may take a while for Slowloris to totally take over high-traffic services, the DDoS attack will cause all legitimate requests to be refused.
Large Payload Transfer Attack [65,66,67]	The current utilization of the Internet of Things involves various applications that require large payload transfer, such as uploading medical data, transmitting audio data from medical devices, detecting vehicle crashes through digital audio, uploading images related to traffic crimes, and uploading binary files generated by industrial machines. Nevertheless, if such data transfers are initiated by devices controlled by hackers, they must be treated as anomalous and potentially malicious.	Complex and large payload transfer can have devastating impact on data transfer messaging protocols namely Constrained Application Protocol (CoAP) [68,69], MQ Telemetry Transport (MQTT) [70,71,72], Extensible Messaging and Presence Protocol (XMPP) [73,74], Advanced Message Queuing Protocol (AMQP) [75], and HTTP [76,77] Anomaly can trigger latency in constant and real-time data transfers. It is crucial to minimize data packet latency and inefficiency without compromising on reliability. It is worth highlighting that a certain amount of inefficiency is necessary for maintainability, flexibility, and testability in an IoT network; without it, these features would be impossible.
Mimicked Device Behavior [78,79]	Behaviors are incorporated into a IoT node Security Profile. For each pattern, the applied protocol will find an associated metric that defines the baseline performance of IoT devices as a whole or as a subset. There are two broad types of actions: behavioral patterns can be detected by either rules or machine learning (ML). ML utilizes past device data to analyze device behavior, whereas rules lets the administrator specify the device behavior. Both machine learning and rule-based thresholds are viable options for a Security Profile to mimic device behavior. Mimicked Device Behavior Attacks in the IoT include malicious software injection attacks, command and control (C&C) attacks, botnet attacks, data manipulation attacks, and rogue access point attacks.	Device behavior analysis triggered by hackers can lead to revealing critical information and may compromise data related to scalability and reliability of IoT network, user’s private data and device design flaws.
Permissions Manipulation [80,81]	The term “permission” refers to the action of bestowing rights upon a verified individual or entity. Authenticated identities in an IoT environment have their capabilities constrained by fundamental regulations. Devices, mobile apps, online apps, and desktop apps all rely on an authorized identity. The types of Permissions Manipulation Attacks in IoT include privilege escalation, horizontal attacks (i.e., attackers can gain access to accounts with limited permissions and then attempt to escalate privileges to gain more), data leaks, and DoS attacks.	The IoT’s inclination to cache copies of policy implies that it may take a few minutes for an adversary’s alterations to bring an action. Then it might take a few seconds to obtain a connection to a resource once it has been introduced to the network, and it can continue to be available for some time after access has been revoked.
Payload Flooding [82,83]	In communications networks, inundation is a straightforward routing approach in which a source or node broadcasts packets over all available outbound connections. When originating packets (lacking routing data) are sent to entire connected network, a phenomenon known as flooding unfolds. Payload Flooding Attacks in the IoT include malformed flooding, unauthorized message flooding, data flooding, command flooding, and fragmentation attack (i.e., the attacker fragments large messages into smaller pieces and sends them to the IoT device, which can cause the device to become overwhelmed and unresponsive).	A “payload flood” attack aims to overwhelm a system with data, making it impossible to distinguish between legitimate and unauthorized data traffic.
SQLi [84]	One typical method for hackers to break into the data-repository of IoT systems illegally, hack the system, and carry out malicious actions is through SQL injection (SQLi). The attack is carried out by inserting malicious script into an otherwise innocuous database query. SQLi attacks can be categorized into three main types (a) In-Band SQLi attacks, (b) Out-of-Bound SQLi attacks, and (c) Inferential SQLi attacks. The main difference between the three categories of SQLi attacks is the method used to send malicious data. In-Band SQLi attacks send malicious data via an input field or web application, Out-of-Band SQLi attacks send malicious data via a different channel such as an email or file transfer, and Inferential SQLi attacks infer data from the database without directly interacting with it.	In SQLi, the contributor’s input is changed by inserting special characters that change the setting of the SQL query. This situation reinforces the attacker’s goals by tricking the repository into running a malicious script rather than the user’s input. Since SQLi may lead to the exposure of private user information or even provide attackers complete administrator access to a database, it can have far-reaching consequences.
DoS Attack [85,86,87]	A Denial-of-Service (DoS) attack is one in which the target system or network is intentionally overloaded to the point where it cannot serve its intended purpose. The types of DoS attacks in the IoT include, but are not limited to, amplification attacks, reflection attacks, flooding attacks, protocol attacks, and resource exhaustion attacks.	The goal of a denial-of-service attack is to make the target system unusable by overloading it with traffic or delivering it data that causes it to fail. DoS attacks can either overload the target’s system or bring it down entirely. When a server is hit with an overwhelming number of requests at once, it is said to be experiencing a flood attack.
Sybil Attack [88,89,90]	During a Sybil attack, the attacker mimics the identities of several different targets. Communicating to a peer-to-peer network presents this as one of the most significant challenges. By constructing several false identities, it is able to influence the network and exert complete control over it. There are several types of Sybil attacks in IoT, including false identity attacks, false data attacks, resource depletion attacks, and denial-of-service attacks.	Sybil attacks, if successful, can prevent Internet of Things devices from communicating with the network and thereby execute a 51% assault. Such attacks can be averted by the use of direct and indirect node validation.
Sinkhole [91,92,93]	In this type of attack, malevolent nodes spread false information to other adjacent nodes in an effort to gain their trust. By sending their transmissions to the compromised nodes, the legitimate nodes equip them to carry out a variety of cyber-attacks. Some of the types of sinkhole attack include selective forwarding attack, blackhole attack, gratuitous routing attack, and replay attack.	With the intention of diverting traffic away from the main hub, the malicious node poses as the shortest path to the access point. This pulls in nodes from a wider area, not just the immediate vicinity of the sinkhole. The data may then be easily manipulated by the intruder node or sinkhole, which compromises the security of the network. A sinkhole attack might originate either from within the network or from the outside IoT environment.
Sinkhole Attack (*DNS Configured*) [94,95,96]	In a sinkhole attack, a hacked node actively seeks out network packets by spreading the message of its deceptive routing transformation. Alternative attacks, such as node capture attacks, acknowledgement replay attack, and dropped or changed routing tables, can be launched from a sinkhole attack. *DNS Sinkholing*, on the other hand, is a method of user protection that involves redirecting DNS queries that are intended for known harmful or undesired domains to a controlled, fake IP address.	An Internet of Things sinkhole attack is one that intentionally slows down or stops an entire network by delivering false routing data. Disrupting communications, denying users access to services, and even launching more assaults on the network are all possible outcomes. In addition, a sinkhole attack may be used to steal information from a network, including IP addresses, data packets, and user passwords. The two most popular forms of sinkhole attacks are blackhole attacks and grayhole attacks. In a blackhole attack, the adversary does not pass along any information it obtains. In a grayhole attack, the malicious node selectively suppresses packets while allowing others to get through.
Blackhole Attack [97,98]	Black hole attacks occur when a router stops forwarding relevant data. A router’s settings can be tweaked such that it establishes a direct connection to any other node on the IoT network at no additional expense. Therefore, all network traffic will be sent to specific routers. The router can also exhibit false failure under certain conditions. Some common types of blackhole attacks in IoT are routing-based blackhole attacks, selective data forwarding attack, false data injection attack, timing attack, reflection attack, and resource consumption attack.	When a malicious node alters the standard operation of the routing protocol, the vulnerable node continues to believe that it has a good route and continues sending data to its intended recipient. An originating node will send out RREQ (route request) to all its communication range as part of the route-finding procedure. When an adversary gets such a request, it will send a RREP (route reply) packet to the originating node, with a high identifier and a low hop count of 1. Data is rendered inaccessible, because the intruder node, upon receiving these packets, discards them without forwarding them to the intended IoT node.
Traffic Analysis Attack [99,100,101]	When an attacker targets an IoT network, they potentially seek to conduct a traffic analysis attack by intercepting or monitoring data sent and received between connected devices. Common types of traffic analysis attacks include eavesdropping [102,103], footprinting [104], network scanning [105,106], and traffic redirection [107].	These exploits have the potential to learn the type of device that is linked to a network, to analyze user behavior, and to extract private information. To counter these threats, enterprises should use an Intrusion Detection System to keep tabs on network activity and implement encryption and robust authentication to safeguard their IoT networks. Researchers should also implement secure protocols such as HTTPS to encrypt data transferred between IoT gadgets.
Man-in-the-Middle Attack (MITM) [108]	A man-in-the-middle attack is a type of cyberattack that occurs in the context of the IoT, in which an attacker intercepts and alters data that is transmitted between two endpoints. This is possible because many IoT devices and infrastructures lack sufficient security measures, making them susceptible to such attacks. The attacker positions himself as the “man in the middle” and has the ability to modify and monitor the data that is being exchanged between the two systems or devices. There are many types of MITM attacks that attackers can use to exploit insecure applications and user data. Some of the most common types of MITM attacks include rogue access point attacks [109], address resolution protocol (ARP) spoofing [110], domain name system (DNS) spoofing [111], session hijacking [112], and SSL/TLS interception [113].	Passwords and critical information are only two examples of the kinds of private data that might be compromised by this kind of cyberattack. The attacker can take charge of the device or system, changing data or issuing malicious commands. In order to stop man-in-the-middle attacks in the IoT, it is essential that all sensors are protected and that all data sent between them is encrypted. In addition, only approved respondents should be provided access to the system; hence, robust authentication mechanisms such as two-factor authentication are highly recommended.

**Table 3:** Limits for detecting anomalies in data.

		IoT Anomaly Detection	Data Security and Consequence Administration Procedure (DSCAP)	Intrusion Detection, Management and Prevention System (IDMPS)	Firewall
	
**Objective**		Physical and Service security	Physical and Service security	Physical and Service security	Physical and Service security
		System and interconnected network supervision	System and interconnected network supervision	Access Management	
		Guaranteeing IoT/IIoT/IoMT accessibility and throughput			
**Scope**		Monitoring and evaluation of the active state of “Operational Technology (OT) networks” that are complicated and have predictable communication techniques	Surveillance of intricate IoT network infrastructures.	Governance of permissions	IoT edge ^1^ security and monitoring
		IoT Control Systems and OT settings are the primary focus, with smart IT infrastructure as a secondary consideration.			
		As a component of a defensive strategy, Tier-based security protects against (persistent, unknown) external attacks.			
**Purpose**		Identification of any deviations from the norm in information exchange (identified and unfamiliar)	An extended inspection of the framework of data transmission	Intercepting internal and external attacks based on their characteristic patterns (identifiers).	Neutralization of identified external anomalies.
		Accessibility and availability in the digital realm	Systems’ security vulnerabilities are being identified		
		A comprehensive evaluation of the communication medium			
		Governance of IoT Assets			
**Characteristics**		Assessment of identified abnormalities’ risk levels	Extensive policy framework	The efficacy of the defense largely dependent on the anomaly-signature repository.	Defense effectiveness is tied to the availability of a comprehensive database of threats.
		Wide-ranging scenario filtering options		Recurrent false-positive and false-negative identification	
**Requirements**		A non-proactive source of information	Real-time data input	Obliges specialized technical understanding.	Consistently implement and upgrade the policy
		Need for specific setup and data hosting	Significant constraints for setup and data handling		
			Necessitates specialized, in-depth understanding of environment ruleset and data requirements.		

^1^ In the IoT, an edge device refers to a computing device that is located close to the edge of a network. These devices are responsible for collecting, processing, and transmitting data from various IoT devices or sensors to the cloud or other centralized servers. Edge devices can take many forms, including smartphones, tablets, laptops, microcontrollers, and specialized IoT gateways. Edge devices are crucial in IoT systems as they help reduce the amount of data that needs to be transmitted over the network to centralized servers or the cloud. This can help to improve the efficiency and speed of data processing, reduce latency, and lower the costs associated with transmitting large amounts of data over the network. Moreover, edge devices often have local storage and processing capabilities, which can help to ensure data availability and continuity in case of network failures or disruptions.

**Table 4:** Access control models and methods for the Internet of Things: an analysis. 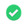 fully triumphed, 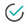 partially triumphed, 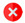 not triumphed.

	Scalability	Process-Centric	Distribution	Data Privacy	Device Imposed Policies	Security	Access Model/Method
Attribute-based [114,115,116]	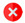	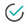	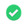	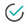	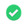	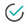	Decentralized access control management application
Capability-based [117,118,119]	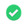	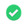	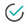	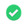	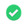	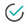	Blockchain driven privacy assurance.
Rule-based [120,121,122]	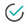	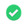	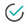	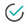	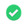	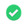	Prioritize sensing and communicating nodes as per their role
LACS [123]	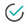	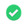	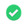	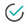	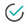	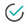	Authorizing Fog nodes based on caching services,
DeCoNet [124]	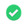	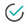	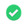	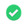	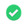	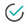	Cluster reachability-distance based utilization of thinning operation to enforce Access validation.
SDSM [125]	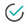	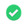	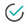	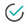	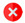	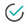	Enforcement of combination of blockchain and ciphertext-based attribute cryptography
SUACC-IoT [126]	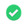	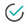	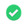	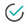	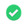	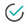	Capability-driven authorization system for IoT devices with limited resources.
OAC-HAS [127]	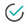	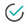	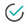	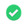	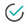	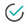	Fog-cloud computing outsourcing verification to avoid access structure data leaks

## Data Availability

Not applicable.
